# Projecting the risk of mosquito-borne diseases in a warmer and more populated world: a multi-model, multi-scenario intercomparison modelling study

**DOI:** 10.1016/S2542-5196(21)00132-7

**Published:** 2021-07-07

**Authors:** Felipe J Colón-González, Maquins Odhiambo Sewe, Adrian M Tompkins, Henrik Sjödin, Alejandro Casallas, Joacim Rocklöv, Cyril Caminade, Rachel Lowe

**Affiliations:** a Centre for the Mathematical Modelling of Infectious Diseases, London School of Hygiene & Tropical Medicine, London, UK; b Centre on Climate Change and Planetary Health, London School of Hygiene & Tropical Medicine, London, UK; c Tyndall Centre for Climate Change Research, School of Environmental Sciences, University of East Anglia, Norwich, UK; d Department of Public Health and Clinical Medicine, Section of Sustainable Health, Umeå University, Umeå, Sweden; e Abdus Salam International Centre for Theoretical Physics, Trieste, Italy; f Heidelberg Institute of Global Health, University of Heidelberg, Heidelberg, Germany; g Department of Livestock and one Health, Institute of Infection, Veterinary and Ecological Sciences, University of Liverpool, Liverpool, UK

## Abstract

**Background:**

Mosquito-borne diseases are expanding their range, and re-emerging in areas where they had subsided for decades. The extent to which climate change influences the transmission suitability and population at risk of mosquito-borne diseases across different altitudes and population densities has not been investigated. The aim of this study was to quantify the extent to which climate change will influence the length of the transmission season and estimate the population at risk of mosquito-borne diseases in the future, given different population densities across an altitudinal gradient.

**Methods:**

Using a multi-model multi-scenario framework, we estimated changes in the length of the transmission season and global population at risk of malaria and dengue for different altitudes and population densities for the period 1951–99. We generated projections from six mosquito-borne disease models, driven by four global circulation models, using four representative concentration pathways, and three shared socioeconomic pathways.

**Findings:**

We show that malaria suitability will increase by 1·6 additional months (mean 0·5, SE 0·03) in tropical highlands in the African region, the Eastern Mediterranean region, and the region of the Americas. Dengue suitability will increase in lowlands in the Western Pacific region and the Eastern Mediterranean region by 4·0 additional months (mean 1·7, SE 0·2). Increases in the climatic suitability of both diseases will be greater in rural areas than in urban areas. The epidemic belt for both diseases will expand towards temperate areas. The population at risk of both diseases might increase by up to 4·7 additional billion people by 2070 relative to 1970–99, particularly in lowlands and urban areas.

**Interpretation:**

Rising global mean temperature will increase the climatic suitability of both diseases particularly in already endemic areas. The predicted expansion towards higher altitudes and temperate regions suggests that outbreaks can occur in areas where people might be immunologically naive and public health systems unprepared. The population at risk of malaria and dengue will be higher in densely populated urban areas in the WHO African region, South-East Asia region, and the region of the Americas, although we did not account for urban-heat island effects, which can further alter the risk of disease transmission.

**Funding:**

UK Space Agency, Royal Society, UK National Institute for Health Research, and Swedish Research Council.

## Introduction

Mosquito-borne disease transmission depends on complex interactions between the environment and the susceptibility, exposure, and adaptive capacity of populations. Variation in climatic factors, including temperature and precipitation, modulate the spatiotemporal distribution of vectors, hosts, and pathogens. Climate change has increased concerns that mosquito-borne disease transmission will intensify through increased vector survival and biting rates, increased replication of pathogens within vectors, shorter reproduction rates, and longer transmission seasons.^1^ The importance of climate change compared with other disease determinants, such as globalisation and urbanisation, remains under debate.

Malaria and dengue, the most important mosquito-borne global threats, are expanding their spatial range, gradually emerging in previously unaffected areas, and re-emerging in areas where they had subsided for decades.^2^, ^3^ Malaria is shifting towards higher altitudes, including the African highlands where climate suitability for transmission increased by about 30% between 2012 and 2017 compared with a 1950 baseline.^3^ Urbanisation trends are associated with increasing dengue risk.^4^ Although differential effects of climate change with altitude and urbanisation have been previously discussed,^3^, ^4^, ^5^ they have not been quantified globally for different altitudes and levels of urbanisation. We stratify future projections of malaria and dengue transmission risk by altitude and population density (as a proxy for urbanisation) for four representative concentration pathways (RCPs) and three shared socioeconomic pathways (SSPs) using a multi-model approach. Building on previous work, we investigate changes in the length of the transmission season (LTS) and the additional population at risk (PAR) to provide policy makers with relevant information to prepare appropriate strategies to build resilience to major mosquito-borne diseases in a warmer and more urbanised world.

**Research in context**

**Evidence before this study**

Malaria and dengue are the most important mosquito-borne disease threats globally. Temperature, rainfall, and humidity influence the spread and the length of transmission season for both diseases. Climate change is expected to increase the risk of malaria in tropical highland areas and to allow dengue to expand into previously unaffected temperate regions. The potential effects of climate change on the transmission of these diseases has not been investigated across different elevations or amounts of urbanisation. We searched PubMed on March 3, 2021, using the terms “malaria”, “dengue”, “model”, “projection”, and “climate change” for papers published between Jan 1, 1990, and March 3, 2021. Although some studies projected changes in these diseases using a suite of emission and socioeconomic scenarios, most studies used a single socioeconomic scenario and focused on a single disease. No studies have used integrated multi-model multi-scenario frameworks to simultaneously assess changes in the length of the transmission season or population at risk for these two major vector-borne diseases over altitudes and urbanisation.

**Added value of this study**

We used an integrated multi-model multi-scenario framework to simultaneously assess the impact of climate change on two different vector-borne diseases over different elevations and amount of urbanisation. We used six emission and socioeconomic scenario combinations and six disease models to quantify uncertainty in our estimates for different elevations and population density (a proxy for urbanisation) for each WHO region. By the end of the century, tropical highland areas in the African region, the Eastern Mediterranean region, and the region of the Americas might experience increases of about 1·6 additional climatically suitable months for malaria transmission (relative to 1970–99). Tropical lowland areas in the Western Pacific region and the Eastern Mediterranean region will show the greatest increase in climatic suitability for dengue transmission (4·0 additional months). The population at risk of both diseases is predicted to increase by up to 4·7 additional billion people, particularly in lowlands and urban areas.

**Implications of all the available evidence**

The predicted increases in climatic suitability and population at risk of malaria and dengue highlight the importance of emission reductions to limit climate change and increased surveillance in potential hotspot areas to monitor disease occurrence and plan adaptation interventions in a warmer and more urbanised world. These actions are particularly important in transmission fringes, where public health systems might be unprepared to control and prevent these diseases.

Malaria is a life-threatening disease caused by protozoans of the genus *Plasmodium*. Five species cause disease in humans, but two of them, *Plasmodium falciparum* and *Plasmodium vivax*, pose the biggest threat, with *P falciparum* being responsible for about 97% of all global cases (∼230 million).^6^ About 93% of all cases occur in sub-Saharan Africa, where malaria is disproportionately linked to inequality and poverty.^6^ Malaria is transmitted between humans by female *Anopheles* mosquitoes, which are mainly found in tropical and subtropical areas, although some species could be found at higher latitudes. The main African vector *Anopheles gambiae* is highly anthropophilic, can feed and rest indoors and outdoors, and likes to feed at night.^7^ *Anopheles* mosquitoes are more ubiquitous in rural areas, although there are growing concerns about urban and peri-urban malaria because of the establishment of *Anopheles stephensi* in eastern Africa.^8^, ^9^

The spatial range of malaria has largely decreased because of human intervention over the past five decades.^10^ The global incidence of malaria declined during 2010–18, except for the WHO Eastern Mediterranean region and Western Pacific region, which saw a slight increase in incidence in that period, and the region of the Americas, where a moderate increase in incidence occurred because of, for example, an unprecedented tenfold increase in cases in Venezuela, from 41  749 cases in 2007 to 411 586 in 2017.^11^

Dengue is the fastest-growing mosquito-borne viral disease in the world, and has substantially expanded its spatial range in the past 60 years.^4^ The disease is endemic in more than 120 countries, and is considered a global health priority. Most cases occur in the WHO region of the Americas, the South-East Asia region, and the Western Pacific region. Dengue is transmitted between humans by female *Aedes* mosquitoes. The two main dengue vectors are *Aedes aegypti* and *Aedes albopictus*. Both species are highly anthropophilic and often compete for the same habitats.^12^ *Aedes* mosquitoes are typically found in urban and peri-urban regions, although *Ae albopictus* are also found in rural environments.^13^ *Ae albopictus* has a cooler thermal optimum (26·4°C) than *Ae aegypti* (29·1°C), and has the ability to diapause over winters.^1^ Consequently, *Ae albopictus* is frequently found in temperate zones. Both species prefer to lay eggs in human-made containers and feed during daytime, particularly at dawn and dusk.

Climate limits the spatial range, timing, and magnitude of malaria and dengue transmission through its effects on vectors and pathogens.^1^ Multiple physiological traits of vectors (eg, biting and development rates) and pathogens (eg, extrinsic incubation period) increase almost exponentially with rising temperatures up to a thermal optimum before declining.^1^ Precipitation shows a similar relationship, with rising precipitation increasing the creation of mosquito breeding sites up to a maximum, after which flooding or flushing could destroy them.^14^ Droughts leading to water shortages can foster the creation of breeding sites by increasing water storage.^15^ Because *Ae aegypti* often breeds indoors, the creation of breeding sites might be exclusively driven by human-driven water storage in some areas. The effects of humidity on dengue and malaria vectors are less understood. Research suggests that high humidity shortens the incubation and blood-feeding intervals of mosquitoes.^16^

Climate change could increase malaria and dengue transmission because of an increased spatial range and length of the transmission season, placing a greater proportion of the global population at risk.^5^, ^17^, ^18^, ^19^ Research suggests a substantial continental expansion, and shifts towards higher altitudes for both diseases. There is disagreement as to whether these shifts will lead to greater disease burden.^10^

Impact models for mosquito-borne diseases are seldom compared to assess the combined effects of climate change on health. Inconsistency in the baseline and projection periods, climate scenarios, modelling methods, and geographical scale between studies make comparisons difficult. The InterSectoral Impact Model Intercomparison Project (ISI-MIP) is a community-driven initiative offering a consistent framework for cross-sectoral modelling of the effects of climate change. ISI-MIP provides bias-corrected climate inputs on a 0·5 × 0·5 degree global grid and daily resolution for four general circulation models (GCMs), four RCPs, and five SSPs^20^ to derive risk estimates. We used the ISI-MIP framework to simultaneously assess changes in the LTS and PAR of malaria and dengue at different altitudes and urbanisation levels. We explore several emission and socioeconomic scenarios and several disease models to assess uncertainty in our estimates. The aim of this study was to quantify the extent to which climate change will influence the LTS season and estimate the PAR of mosquito-borne diseases in the future, given different population densities across an altitudinal gradient.

## Methods

### Model inputs: climate data for the experiments

Our study was designed as a multi-model multi-scenario intercomparison modelling study. Bias-corrected global daily mean surface temperature (in K), total precipitation (in kg/m^2^ per second), and relative humidity (in %) data were retrieved from the ISI-MIP database on a 0·5 × 0·5 degree latitude–longitude grid. We obtained data for four GCMs (HadGem2-ES, IPSL-CM5A-LR, MIROC-ESM-CHEM, and GFDL-ESM2M) across four RCPs (arranged from the most conservative to business-as-usual: RCP2·6, RCP4·5, RCP6·0, and RCP8·5) to represent several radiative forcings. RCPs are possible scenarios of the combined effect of greenhouse gases and other factors that might influence climate. RCPs are named on the basis of their end-of-century radiative forcing relative to preindustrial conditions (eg, RCP2·6 indicates a 2·6 W/m^2^ increase). Historical climate data were collated for the period of 1951–2005 and future data for the period of 2006–99, to give continuity to our historical runs, in line with the Coupled Model Intercomparison Project Phase 5.

### Model inputs: population data

We obtained global gridded annual population counts on a 0·5 × 0·5 degree latitude–longitude grid from population studies for the historical (1951–2005) and future (2006–99) time periods.^21^ We obtained future projections for three socioeconomic scenarios (low challenges to mitigation and adaptation in SSP1; medium challenges to mitigation and adaptation in SSP2; and high challenges to mitigation with low challenges to adaptation in SSP5). Population density for each SSP (per km^2^) was calculated by dividing population counts by the surface area of each cell.

### Malaria disease models

We used three malaria models with varying amounts of complexity (appendix pp 1–4). All malaria models were parameterised for *P falciparum* and *An gambiae*. The Lancet Countdown malaria indicator (LCMI) is a threshold-based model that tracks global changes in the climatic suitability for malaria.^3^ Climatic suitability is defined as a coincidence of precipitation accumulation greater than 80 mm, an average temperature of 18–32°C, and relative humidity greater than 60%.^22^ The combined values are an indication of the lower limit for *P falciparum* transmission. The Liverpool Malaria Model (LMM_R_0_)^23^ is a simplified, steady-state solution version of the weather-driven, mathematical–biological Liverpool Malaria Model.^24^ Monthly adult mosquito populations are proportional to precipitation in the previous month. Adult mosquito populations are then combined with the biting rate, sporogonic cycle length, and survival probability calculated using mean temperature to derive the basic reproduction number, R_0_. VECTRI^25^ is a mathematical malaria model that accounts for the effects of daily temperature and precipitation on the development cycle of *P falciparum* and *An gambiae*. VECTRI accounts for the effects of temperature in the sporogonic and gonotrophic cycles, and the mortality rate of adult mosquitoes. The effects of precipitation on transmission are represented by a surface pool hydrology model. VECTRI also accounts for human population density in the calculation of malaria risk. The model includes immunity in the host population and uses a genetic machine learning algorithm.^26^

### Dengue disease models

We used two mechanistic models and one statistical model for dengue. The first two models consist of vectorial capacity models including basic vector to human interactions coupled with stage-structured data-driven dynamic models to describe the population dynamics of *Ae aegypti* (UMEÅ-*aegypti*) and *Ae albopictus* (UMEÅ-*albopictus*).^27^, ^28^, ^29^ These models account for the effects of daily temperature, precipitation, and daylight length in the ecological processes of mosquito populations and for the spatiotemporal dynamics of mosquito populations. The statistical dengue model (DGM) is a generalised additive mixed model that simulates dengue incidence as a function of temperature, precipitation, relative humidity, and population density. Delayed effects and non-linear relationships are assumed for the climatic effects. An earlier version of the model was used to estimate the potential impacts of varying amounts of global warming on dengue incidence across Latin America.^18^

### Model metrics

LTS and PAR were calculated for each grid cell using a 0·5 × 0·5 degree latitude–longitude grid. We defined LTS as the number of suitable transmission months per year. PAR is the total population in a grid cell having at least one suitable month in the same year. LTS and PAR were calculated for each GCM, and for six RCP and SSP combinations (appendix p 1). For any given month, LTS is either 1 or 0. For LCMI, LTS was equal to 1 in a month if total precipitation was greater than 80 mm per month, average temperature was 18–32°C, and relative humidity was greater than 60%.^22^ For LMM_R_0_, LTS was equal to 1 if R_0_ was higher than 1; otherwise, LTS was equal to 0. For VECTRI, LTS was equal to 1 in a month if the daily entomological inoculation rate was higher than 0·1.^5^ UMEÅ-*aegypti* and UMEÅ-*albopictus* used a similar criterion but with a logistic function to allow for a 90% uncertainty range in the classification of R_0_. For DGM, LTS was equal to 1 if the incidence rate exceeds 1 case per 100 000 inhabitants.

### Role of the funding source

The funders of this study had no role in study design, data collection, data analysis, data interpretation, or writing of the report.

## Results

**Figure 1:**
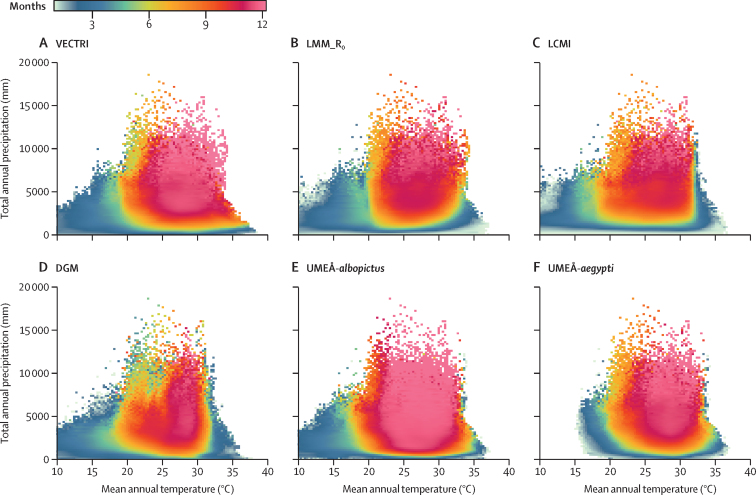
Relationship between LTS (months), temperature, and precipitation LTS relationship with mean annual temperature and total annual precipitation for the VECTRI (A), LMM_R_0_ (B), LCMI (C), DGM (D), UMEÅ-*albopictus* (E), and UMEÅ-*aegypti* (F) models. Values were binned linearly (n=100 bins). The computation was based on all spatial points and annual time steps for each disease model. For future scenarios only SSP2 (medium challenges to mitigation and adaptation) was considered. DGM=dengue model. LCMI=The Lancet Countdown malaria indicator. LMM_R_0_=The Liverpool Malaria Model. LTS=length of the transmission season. SSP=shared socioeconomic pathway.

Associations between LTS, temperature, and precipitation were non-linear and unimodal with greater LTS values at intermediate temperatures and amounts of precipitation (figure 1). The optimal climate conditions for year-round malaria transmission (for which LTS=12) were observed between 20·9°C and 34·2°C (mean 29·4°C, SE 2·6) and between 5050·5 mm and 15 959·6 mm precipitation per year (mean 10 968·9, SE 1974·7). Increases in malaria LTS in the future projections (relative to the period 1970–99) were constrained to temperatures lower than 25°C and decreases in malaria LTS to temperatures higher than 35°C (appendix p 5). Changes in both directions occurred between 0 mm and 5000 mm precipitation, although VECTRI predicted decreases in LTS with total rainfall lower than 2500 mm per year.

Optimal conditions for dengue transmission were observed between 18·5°C and 33·0°C (mean 28·2, SE 2·7) and between 6262·6 mm and 18 585·9 mm per year (mean 10 979·7, SE 1908·2). The thermal minimum of the non-vector-specific DGM model and UMEÅ-*albopictus* model was 10·0°C, whereas the minimum for the UMEÅ-*aegypti* model was 14·8°C. Increases in dengue LTS in the future projections were observed at temperatures lower than 35°C, and decreases in dengue LTS with temperatures higher than 35°C and total rainfall lower than 2000 mm per year (appendix p 5).

**Figure 2:**
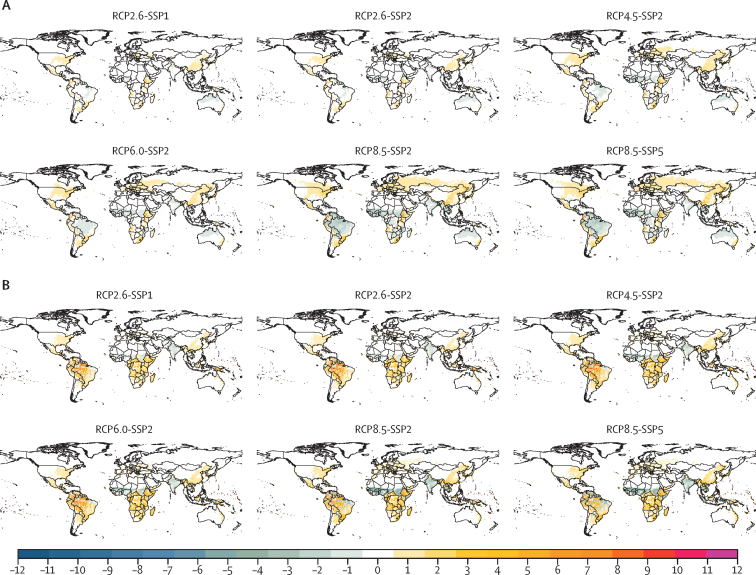
Simulated changes in LTS Ensemble mean of the simulated changes in LTS for malaria (A) and dengue (B) across six emission and socioeconomic scenario combinations averaged over the period 2070–99 relative to the period 1970–99. SSPs include SSP1 (low challenges to mitigation and adaptation), SSP2 (medium challenges to mitigation and adaptation), and SSP5 (high challenges to mitigation with low challenges to adaptation). Blue colours indicate decreases in LTS and orange or pink colours indicate increases in LTS (months). LTS=length of the transmission season. RCP=representative concentration pathways. SSP=shared socioeconomic pathways.

Year-round malaria and dengue transmission (LTS=12) was simulated in tropical regions over the period 2006–99. The LTS of both diseases gradually shortened with latitude to 1–2 months over epidemic transmission fringes, in line with the thermal performance (figure 1). The spatial range of both diseases gradually increased with time towards temperate zones. Expansion to temperate zones was more modest for dengue than for malaria (appendix pp 6–8). The LTS of malaria and dengue transmission increased over time and with emissions in several tropical, subtropical, and temperate areas (appendix pp 9–10). Towards the end of the century, malaria LTS consistently increased across all scenarios over northeastern USA, the Mexican plateau, the Andean region, eastern South America, northern Italy, the Balkans, the horn of Africa, Angola, South Africa, Madagascar, eastern Australia, and several areas in Indonesia and east Asia. Consistent decreases were predicted in the northern half of South America, sub-Saharan Africa, the Indian subcontinent, southeast Asia, and northern Australia (figure 2). Dengue models consistently predicted increases in LTS over northeastern USA, central America, the northern half of South America, sub-Saharan Africa, east Asia, and several areas of southeast Asia. Some increases were detected across continental Europe with the non-vector-specific DGM and UMEÅ-*albopictus* models, but these increases were modest (*<*3 months). UMEÅ-*aegypti* did not predict increases in LTS in Europe. Consistent decreases in dengue LTS were consistently predicted over the Sahel and the Indian subcontinent. For both diseases, there are large geographical areas with no effect, particularly in temperate and arid regions.

**Figure 3:**
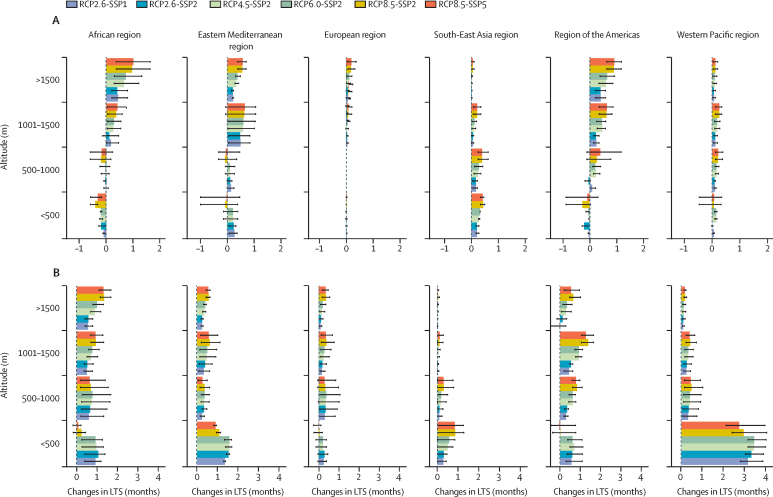
Changes in LTS by altitude Ensemble mean of the simulated changes in LTS for malaria (A) and dengue (B) stratified by altitude, emissions, and socioeconomic scenario combination. The bars indicate the multi-model (three models per disease) ensemble mean for each scenario. Error bars indicate the spread of the predictions. SSPs include SSP1 (low challenges to mitigation and adaptation), SSP2 (medium challenges to mitigation and adaptation), and SSP5 (high challenges to mitigation with low challenges to adaptation). LTS=length of the transmission season. RCP=representative concentration pathways. SSP=shared socioeconomic pathways.

Relative to the period 1970–99, we predict average increases of up to a maximum of 1·6 additional months (mean 0·5, SE 0·03) in malaria LTS in highland areas (altitude >1000 m) in the WHO African region, the Eastern Mediterranean region, and the region of the Americas, where LTS increased both with altitude and emissions towards the end of the century (figure 3). At lower altitudes, models predicted increases of up to a maximum of 1·2 months although, on average, models predicted a decrease in LTS (mean −0·02, SE 0·03). Modest decreases in malaria LTS were predicted with rising altitude across the South-East Asian region from a maximum of 0·5 additional months (mean 0·3, SE 0·02) in lowlands (<500 m) to 0·1 months (mean 0·03, SE 0·01) in highlands (>1500 m). Modest changes were predicted in the European region and the Western Pacific region with increases of up to 0·4 months (mean 0·1, SE 0·01) across both highlands and lowlands. Higher emissions resulted in slightly larger increases in LTS than lower emissions.

Dengue LTS decreased with rising altitude from a maximum of 4·0 additional months (mean 1·7, SE 0·2) in lowlands (<500 m) to a maximum of 1·1 months (mean 0·2, SE 0·02) in highlands (>1000 m) in the Eastern Mediterranean region, the South-East Asian region, and the Western Pacific region by the end of the century. The highest increases were predicted in the Western Pacific region. In the African region, dengue LTS gradually increased with rising altitude from a maximum of 1·4 months (mean 0·6, SE 0·1) in lowlands (<500 m), to a maximum of 0·8 months (mean 1·0, SE 0·1) in highlands (>1500 m). In the region of the Americas, dengue LTS gradually increased with altitude from a maximum of 1·1 months (mean 0·5, SE 0·1) in lowlands (<500 m) to a maximum of 1·7 months (mean 0·6, SE 0·1) in highlands (>1000 m).

**Figure 4:**
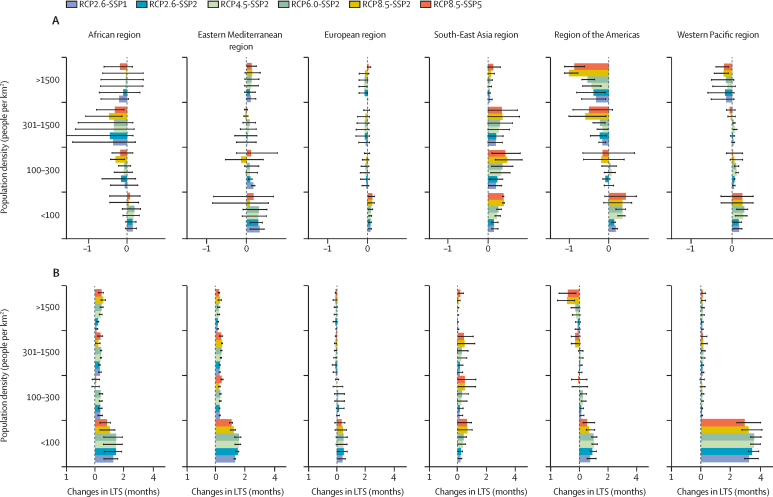
Changes in LTS by population density Ensemble mean of the simulated changes in LTS for malaria (A) and dengue (B) stratified by population density, emissions, and socioeconomic scenario combination. The bars indicate the multi-model ensemble mean for each scenario. Error bars indicate the spread of the predictions. SSPs include SSP1 (low challenges to mitigation and adaptation), SSP2 (medium challenges to mitigation and adaptation), and SSP5 (high challenges to mitigation with low challenges to adaptation). LTS=length of the transmission season. RCP=representative concentration pathways. SSP=shared socioeconomic pathways.

Models predicted consistent decreases in malaria LTS with increasing population density in all regions (figure 4) by the end of the century. On average, malaria LTS decreased from a maximum of 0·7 months (mean 0·2, SE 0·02) in rural areas (<100 people per km^2^) to a minimum of −1·6 months (mean −0·1, SE 0·02) in urban areas (>300 people/km^2^). Uncertainty varied greatly between regions and was larger in Africa. Larger uncertainties were associated with the disease models used.

Dengue LTS decreased with increasing population density in all regions and most scenarios (figure 4) from a maximum of 4·0 months (mean 1·2, SE 0·1) in rural areas (<100 people/km^2^) to a maximum of 1·2 months (mean 0·1, SE 0·02) in urban areas (>300 people/km^2^). Changes in dengue LTS were considerably greater in the Western Pacific region than in other regions.

On average, the PAR of malaria increased with time to about 3·6 billion additional people in 2071, after which time it gradually declined (appendix p 11). We observed similar patterns across all scenarios and impact models, with the largest increases observed in scenario RCP8·5-SSP2, in which PAR increased up to about 4·7 billion additional people between 2071 and 2078. The most conservative estimates were predicted by VECTRI. LMM_R_0_ and LCMI predicted similar values. Similar patterns were predicted for the PAR of dengue (appendix p 11) with a peak at approximately 3·6 billion additional people in 2067, a gradual decline thereafter because of assumptions in the SSP data, and the largest increases (ie, 4·7 billion additional people in 2080) predicted for scenario RCP8.5-SSP2. The highest estimates were predicted by DGM, followed by UMEÅ-*albopictus* and UMEÅ-*aegypti*.

**Figure 5:**
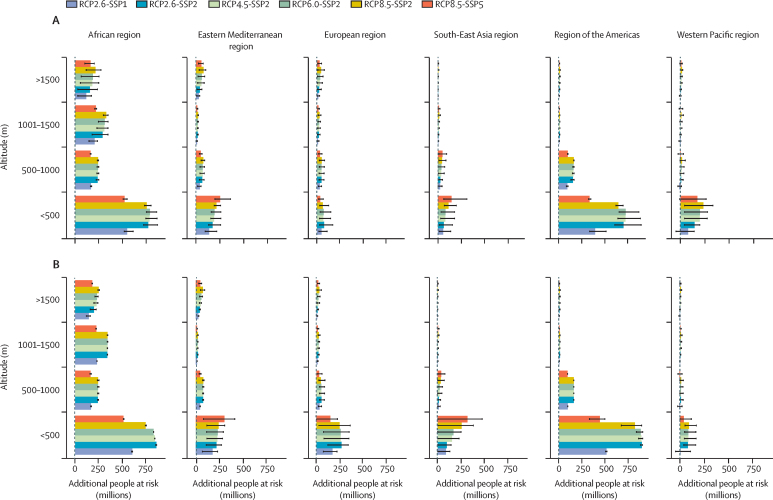
Additional PAR by altitude Ensemble mean of the additional PAR of malaria (A) and dengue (B) stratified by altitude, emissions, and socioeconomic scenario combination. The bars indicate the multi-model ensemble mean for each scenario. Error bars indicate the spread of the predictions. SSPs include SSP1 (low challenges to mitigation and adaptation), SSP2 (medium challenges to mitigation and adaptation), and SSP5 (high challenges to mitigation with low challenges to adaptation). PAR=population at risk. RCP=representative concentration pathways. SSP=shared socioeconomic pathways.

The additional PAR for both diseases gradually decreased with rising altitude across all regions and scenarios (figure 5). The additional PAR of malaria decreased from a maximum of 2·6 billion people (mean 1·8, SE 0·1) in lowlands (*<*500 m across all regions), to a maximum of 0·5 billion people (mean 0·3, SE 0·03) in highlands (>1500 m across all regions). The additional PAR of dengue decreased from a maximum of 2·9 billion people (mean 2·1, SE 0·1) in lowlands (*<*500 m) to a maximum of 0·4 billion people (mean 0·3, SE 0·02) in highlands (>1500 m).

**Figure 6:**
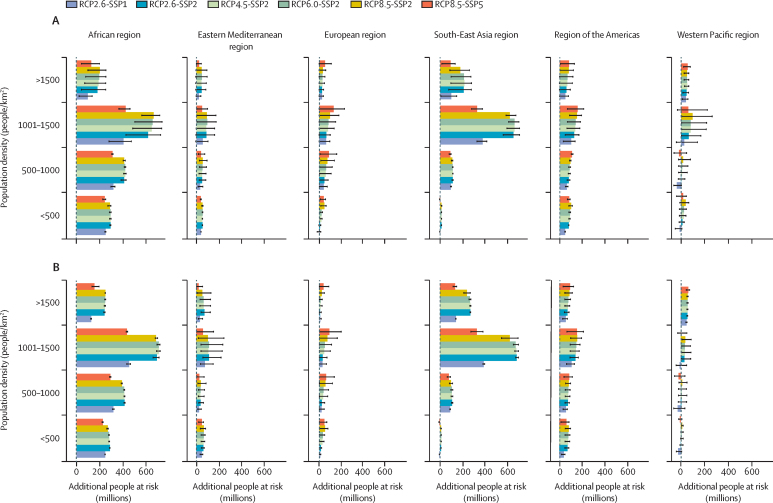
Additional PAR by population density Ensemble mean of the additional PAR for malaria (A) and dengue (B) stratified by population density, emissions, and socioeconomic scenario combination. The bars indicate the multi-model ensemble mean for each scenario. Error bars indicate the spread of the predictions. SSPs include SSP1 (low challenges to mitigation and adaptation), SSP2 (medium challenges to mitigation and adaptation), and SSP5 (high challenges to mitigation with low challenges to adaptation). PAR=population at risk. RCP=representative concentration pathways. SSP=shared socioeconomic pathways.

The additional PAR of both diseases is predicted to be larger in urban areas (*>*300 people per km^2^) across all regions (figure 6). The maximum PAR of malaria was estimated to be a maximum of 736 million additional people (mean 348, SE 38·7) in the African region, followed by the South-East Asia region with a maximum of 704 million additional people (mean 349, SE 38·8), and the region of the Americas with a maximum of 211 million additional people (mean 92, SE 10·0). The maximum PAR of dengue was estimated to be a maximum of 725 million additional people (mean 415, SE 37·6) in the African region, followed by the South-East Asia region with a maximum of 696 million additional people (mean 390, SE 34·7), and the region of the Americas with a maximum of 211 million additional people (mean 103, SE 7·9).

## Discussion

We present a multi-model, multi-scenario, multi-pathogen intercomparison to simultaneously assess the potential impacts of climate change on the future risk of transmission of two major mosquito-borne diseases, stratified by different amounts of elevation and urbanisation. We use consistent baseline and projection periods, and the same model inputs to make between-model comparisons possible.

All impact models showed unimodal responses to temperature, peaking between 18·2 and 36·1°C (mean thermal optimum 28·4°C) and declining at lower and higher temperatures, in agreement with previous studies.^1^ Unimodal relationships were found using both mechanistic and statistical approaches, indicating that our models capture the increased heat stress that vectors show at high temperatures.

Our findings offer strong support for a northward shift of the malaria-epidemic belt in North America, central northern Europe, and northern Asia.^5^ In contrast with studies using spatial modelling approaches,^17^ we predict a northward shift of the dengue-epidemic belt over central northern Europe and northern USA because of increases in suitability, echoing findings from other groups.^19^, ^27^ Thus, epidemiological surveillance and public health responses might need to be done for longer periods, increasing the demand for already scarce resources. We note that these shifts might not translate into increased morbidity if health systems identify and suppress infections.^5^ Our findings are in line with the emergence of autochthonous cases in Europe,^30^ and the increasing presence of *Ae albopictus* and *Ae aegypti* in the region.^31^ Importantly, we show that low-emission scenarios result in lower LTS and PAR estimates than high-emission scenarios. Thus, our findings support the notion that limiting global mean temperature increases well below 2°C can substantially reduce public health risks.^18^

We predict that malaria suitability will gradually increase as a consequence of a warming climate in tropical areas in most tropical regions, especially highland areas in the African region, the Eastern Mediterranean region, and the Americas, as previously estimated.^2^, ^5^, ^32^ Dengue suitability is predicted to increase mostly in lowland areas in the Western Pacific region and the Eastern Mediterranean region, and in highland areas in the Americas, as previously proposed.^17^, ^18^ Shifts towards higher altitudes suggest that outbreaks can occur in areas where people might be immunologically naive and public health infrastructure unprepared. This situation could have major impacts on populations, public health systems, and economies with no previous experience in managing these diseases. At altitudes below 1000 m, climatic and socioeconomic conditions could decrease malaria transmission in Africa even when maintaining current malaria interventions, as previously proposed.^10^

Our simulated decreases in the climatic suitability for malaria in sub-Saharan Africa, and the simulated increases in the climatic suitability for dengue transmission in the region support the hypothesis that climate change could promote a shift from malaria to dengue transmission in sub-Saharan Africa in the future.^32^ Warmer-adapted anopheline species (eg, *An stephensi*) could also invade and replace *An gambiae* populations and continue malaria transmission in sub-Saharan Africa.^32^

We predict increases of about 1·4 billion additional people at risk of malaria and dengue in urban areas in Africa and southeast Asia. Urbanisation is an important driver of mosquito-borne disease transmission, because it enhances the creation of mosquito breeding sites via human-made containers, increases the likelihood of vector–human interactions because of higher population densities, and facilitates spatial spread through the movement of people and goods. We used population density as a proxy for urbanisation. However, we acknowledge that urbanisation is a complex process also involving population shifts from rural to urban areas and ways in which society adapts to this change. Urban areas also provide microclimates that might enhance the development and survival of some mosquito species, such as *An stephensi* and *Aedes* mosquitoes.^32^ Synergies between urbanisation and warmer temperatures could increase the risk of urban malaria from *An stephensi* or promote a shift from malaria to dengue transmission.^32^ However, we acknowledge that our malaria models are not parameterised for *An stephensi*, and so we could not test this hypothesis. We also did not take into account the potential effects of other important drivers, such as human mobility^33^ or interventions that can lead to different risk patterns.

There are several key limitations of this study. Along with human mobility and interventions, we did not consider the effects of socioeconomic development, disease and vector evolution, or the development of more effective drugs and vaccines, all of which could lead to important differences in the amount of risk we simulate. Our estimates are constrained by our selection of GCM and disease models, and the selected combinations of emission and socioeconomic scenarios. Future experiments could incorporate larger model ensembles and scenario combinations to provide a richer view of the uncertainty around our estimates. Simulations of our statistical model were limited by data constraints, highlighting the need for publicly available long-term data sets of sub-national surveillance data. Despite these limitations, this study provides useful information for policy making and public health preparedness.

## Data sharing

All model outputs are available through the Centre for Open Science.

## Declaration of interests

We declare no competing interests.
