
JOURNAL INFORMATION
==============================
NLM Title Abbreviation: Lancet Planet Health
Journal ID: Lancet Planet Health
NLM Journal ID: 101704339

The Lancet. Planetary Health

EISSN: 2542-5196

ARTICLE INFORMATION
==============================
PMCID: PMC8386288
PMID: 34390666
DOI: 10.1016/S2542-5196(21)00207-2
Article ID: S2542-5196(21)00207-2
Article version: 1
Subjects: Corrections

Correction to Lancet Planet Health 2021; 5: e426–35

Publication date: 2021 Aug
Electronic publication date: 2021 Aug 11
Volume: 5
Issue: 8
First page: e504
Last page: e504
Copyright: © 2021 The Author(s). Published by Elsevier Ltd.
Copyright year: 2021
Copyright holder: Elsevier Ltd
License: This is an open access article under the CC BY license (http://creativecommons.org/licenses/by/4.0/).
License URL: https://creativecommons.org/licenses/by/4.0/
Erratum for: Projecting the risk of mosquito-borne diseases in a warmer and more populated world: a multi-model, multi-scenario intercomparison modelling study 5 7 7 7 2021 e404 e414 The Lancet. Planetary Health 10.1016/S2542-5196(21)00132-7 PMC8280459 34245711

==============================
Trinanes J, Martinez-Urtaza J. Future scenarios of risk of Vibrio infections in a warming planet: a global mapping study. Lancet Planet Health 2021; 5: e426–35—In this Article, the email address of the corresponding author has been changed. This correction has been made as of Aug 11, 2021.

