# Supplementary material for: Projecting the risk of mosquito-borne diseases in a warmer and more populated world: a multi-model, multi-scenario intercomparison modelling study
Source: Lancet Planet Health. 2021 Jul 7;5(7):e404–14. doi: 10.1016/S2542-5196(21)00132-7 (PMC8280459; doi:10.1016/S2542-5196(21)00132-7)
Supplement: Supplementary appendix [file mmc1.pdf]

THE LANCET
Planetary Health

Supplementary appendix

This appendix formed part of the original submission and has been peer reviewed. We post it as supplied by the authors.

Supplement to: Colón-González FJ, Sewe MO, Tompkins AM, et al. Projecting the risk of mosquito-borne diseases in a warmer and more populated world: a multi-model, multi-scenario intercomparison modelling study. *Lancet Planet Health* 2021; **5:** e404–14.

Projecting the risk of mosquito-borne diseases in a warmer and more urbanised world: a multi-model multi-scenario intercomparison study

Felipe J. Colón-González, PhD; Maquins Odhiambo Sewe, PhD; Adrian M. Tompkins, PhD; Henrik Sjödin, PhD; Alejandro Casallas, MSc; Prof. Joacim Rocklöv, PhD; Cyril Caminade, PhD; Rachel Lowe, PhD

Supplementary Materials and Methods

Historical climate data for the statistical dengue model

Historical climate data were obtained from the Climate Data Store (https://cds.climate.copernicus. eu/). Hourly near-surface temperature (K) and hourly rainfall flux (kg m^{-2} s^{-1}) were retrieved from the bias corrected near-surface meteorological variables (WFDE5) derived from the fifth generation of the European Centre for Medium-Range Weather Forecasts atmospheric reanalyses (ERA5) on a 0.5 × 0.5 deg latitude-longitude grid for land pixels only for the period January 1999 to December 2017. Monthly relative humidity (%) was obtained for the same period from the ERA5 reanalysis on a 0.25 × 0.25 deg latitude-longitude grid. Hourly data were aggregated at monthly time steps using standard methods in the Climate Data Operators (CDO) software.[1] Spatial aggregation at the province (admin1) level was conducted in R version 3.6.1 using the `raster` R package.[2]

Climate data for the experiments

Bias-corrected mean surface temperature (K), total monthly precipitation (kg m^{-2} s^{-1}), and relative humidity (%) data were retrieved at a 0.5 × 0.5 deg global grid spatial resolution at daily time steps from the ISI-MIP server (https://esg.pik-potsdam.de/projects/isimip/). Data were obtained for four GCM (HadGem2-ES, IPSL-CM5A-LR, MIROC-ESM-CHEM, GFDL-ESM2M) to investigate effects across a wide range of climatic changes. Data were retrieved for each GCM across four RCP emission scenarios (arranged from the most conservative to business as usual: RCP2.6, RCP4.5, RCP6.0 and RCP8.5) to represent a suite of radiative forcings. RCPs are named based on their end-of-century radiative forcing levels. Thus, RCP2.6 indicates a 2.6 W m^{-2} increase in radiative forcing relative to pre-industrial conditions. Climate data were aggregated at monthly time steps for some disease models (i.e., LMM_R_0, LCMI, DGM, UMEÅ-*albopictus* and UMEÅ-*aegypti*) using standard methods in the Climate Data Operators software.[1] Historical climate data were collated for the period 1951–2005, and future scenario data for the period 2006–2099.

SSP-RCP scenario combinations

We explored a suite of RCP-SSP combinations to explore the potential effects of multiple emission and socioeconomic scenarios.[3] Table 2 depicts the scenarios selected in our study. SSP1-RCP2.6 represents the lower end on the range of future radiative forcing pathways albeit with substantial land use change.[3] SSP2-RCP2.6 is also at the lower end of future radiative forcing pathways but its land use and aerosol pathways are not extreme compared to other SSPs.[3] SSP2-RCP4.5 is at the middle of the range of future radiative forcing pathways and combines intermediate socioeconomic vulnerability with intermediate forcing.[3] SSP2-RCP6.0 is also at the medium range of future radiative forcing pathways combined with an intermediate socioeconomic scenario.[3] SSP5-RCP8.5 represents the high end of the range of future forcing pathways combined with an intermediate socioeconomic scenario.[3] SSP5-RCP8.5 represents the high end of the range of future radiative forcing pathways, and is combined with the only socioeconomic scenario able to produce a radiative forcing of 8.5 W m^{-2} by 2100.[3]

Table A2: RCP-SSP scenario matrix illustrating. Each cell indicates a combination between a representative concentration pathway and a shared socioeconomic pathway (SSP)

	**RCP2.6**	**RCP4.5**	**RCP6.5**	**RCP8.5**
**SSP1**	X			
**SSP2**	X	X	X	X
**SSP5**				X

Epidemiological data for calibrating VECTRI

The VECTRI model is calibrated using a machine learning technique based on a constrained genetic algorithm outlined in.[4] An ensemble of 80 gridded simulations (where the size of the grid is defined by that of the climate input) are conducted for the period of 1980 to 2012 at daily time steps for the Ethiopian region, with each simulation using a perturbed value for 18 key model parameters that govern the mosquito and parasite life cycle, such as the gonotrophic and sporogonic cycle length and the vector mortality for example. Each simulation is assigned a probability related to the simulations' skill level and a likelihood of the parameter settings. The model skill is determined by the probability of finding the parasite ratio recorded at over 200 field observation sites within Ethiopia collated for the Malaria Atlas Project,[5] comparing the simulation at the same locations and dates of the observations. The penalty function is instead the probability of the model parameter settings given the prior assessment of their default value and uncertainty. This latter cost-function contribution penalizes models that employ parameter settings that lie far outside their assessed probable range. The settings in the *next generation* of models are adopted according to this probability, while a small number undergo mutation to allow the model to explore the parameter space.

Epidemiological data for the statistical dengue model

Monthly dengue cases for the period January 2000 to December 2017 at the province (i.e. admin1) level were obtained from a suite of sources (Table 1) for Argentina, Brazil, Cambodia, Colombia, Ecuador, El Salvador, Indonesia, Malaysia, Mexico, Nicaragua, Panama, Paraguay, Peru, Philippines, Singapore, Thailand, and Vietnam. Epidemiological data for Cambodia and Vietnam could only be obtained at the national and annual levels for the period 2011-2017. Annual dengue data were linearly downscaled at the province and disaggregated at the monthly levels using the historical mean monthly proportion of cases for each province following Gaffin et al, (2004).[6]

Table A1: Sources of the epidemiological data used to train the statistical dengue model.

COUNTRY	PERIOD COVERED	SOURCE
**Argentina**	Jan 2004 – Oct 2004	https://www.tycho.pitt.edu/data/#datasets
**Argentina**	Jan 2011 – Dec 2017	https://www.argentina.gob.ar/salud/epidemiologia/boletines2011
**Brazil**	Jan 2010 – Dec 2017	http://datasus.saude.gov.br/acesso-a-informacao/doencas-e-agravos-de-notificacao-de-2007-em-diante-sinan/
**Cambodia**	Jan 2000 –Dec 2010	https://www.tycho.pitt.edu/data/#datasets
**Cambodia**	Jan 2011 – Dec 2017	http://cdcmoh.gov.kh/surveillance/camewarn
**Colombia**	Jan 2007 – Dec 2017	https://www.minsalud.gov.co/salud/Paginas/BOLETINESEPIDEMIOLOGICOS.aspx´
**Ecuador**	Jan 2013 – Dec 2017	https://www.salud.gob.ec/gaceta-epidemiologica-ecuador-sive-alerta/
**El Salvador**	Jan 2000 – Dec 2010	https://www.tycho.pitt.edu/data/#datasets
**El Salvador**	Jan 2011 – Dec 2017	https://www.salud.gob.sv/boletines-epidemiologicos-2020/
**Indonesia**	Jan 2000 – Dec 2010	https://www.tycho.pitt.edu/data/#datasets
**Indonesia**	Jan 2011 – Dec 2017	https://pusdatin.kemkes.go.id/folder/view/01/structure-publikasi-data-pusat-data-dan-informasi.html
**Malaysia**	Jan 2000 – Dec 2010	https://www.tycho.pitt.edu/data/#datasets
**Malaysia**	Jan 2011 – Dec 2017	https://www.data.gov.my/data/en US/dataset/penyakit-denggi/resource/276e6916-19a0-49da-8b64-1cd4b70ad082
**Mexico**	Jan 2000 – Dec 2017	https://epidemiologia.salud.gob.mx/anuario/html/anuarios.html
**Nicaragua**	Jan 2000 – Apr 2005	https://www.tycho.pitt.edu/data/#datasets
**Panama**	Mar 2003 – Apr 2005	https://www.tycho.pitt.edu/data/#datasets
**Panama**	Jan 2015 – Dec 2017	https://www.mida.gob.pa/direcciones/direcciones nacionales/direcci-n-nacional-de-salud-animal/boletines-epidemiol-gicos.html

**Paraguay**	Mar 2012 – Dec 2017	http://www.vigisalud.gov.py
**Peru**	Mar 2004 – Dec 2017	https://www.dge.gob.pe/portalnuevo/publicaciones/boletines-epidemiologicos/
**Philippines**	Jan 2000 – Dec 2010	https://www.tycho.pitt.edu/data/#datasets
**Philippines**	Mar 2013 – Dec 2017	https://www.doh.gov.ph/statistics
**Singapore**	Jan 2000 – Dec 2010	https://www.tycho.pitt.edu/data/#datasets
**Singapore**	Jan 2001 – Dec 2017	https://www.moh.gov.sg/resources-statistics/infectious-disease-statistics/2018/weekly-infectious-diseases-bulletin
**Thailand**	Jan 2000 – Dec 2010	https://www.tycho.pitt.edu/data/#datasets
**Thailand**	Jan 2011 – Dec 2017	https://wesr.doe.moph.go.th
**Vietnam**	Jan 2000 – Dec 2010	https://www.tycho.pitt.edu/data/#datasets
**Vietnam**	Jan 2011 – Dec 2017	http://soyte.hatinh.gov.vn/tin-tuc-su-kien/thong-tin-y-te/kiem-tra-giam-sat-sot-xuat-huyet-tai-xa-ky-thu-huyen-ky-anh.html

Technical details of the Umeå models

The R_0 values derived from the UMEÅ-*albopictus* and UMEÅ-*aegypti* models follow from a vectorial capacity (VC) computation, which expresses the average daily rate of subsequent cases in a susceptible population resulting from one infected case. Computations were estimated as follows:

$$VC = -\frac{ma^2 b_m p^n}{\ln(p)}$$

where a is the average vector biting rate, b_m is the probability of vector infection and transmission of virus to its saliva, n is the extrinsic incubation period, m denotes the female mosquito to human population ratio, and p is the daily survival probability. All parameters are temperature dependent and are further described in Rocklöv and Tozan (2019).[7] The female mosquito populations are estimated as potential vector abundance for *Aedes aegypti* and *Aedes albopictus*.[8] Stage-structured data-driven dynamical models were used to describe the population dynamics of *Ae. aegypti* (UMEA-*aegypti*) and *Ae. albopictus* (UMEA-*albopictus*).[8,9]

These models account for the effects of temperature, precipitation and day-light length on the ecological processes of the mosquito-population dynamics. Temperature and precipitation inputs were derived from climate data (see subsection Climate data for the experiments). Day-light length was computed using a standard periodic function depending on latitude [9, 10]. For an in-depth model-description of the *Ae. aeqypti* model, see Liu-Helmersson et al., (2019),[8] and for the *Ae. albopictus* model, see Di Sierra et al., (2018).[9]

VC depends only on vector biology and is intrinsically related to the basic reproduction number (R_0) for vector-borne diseases, which is the expected number of hosts to be infected by a single infected host in a susceptible population. R_0 is formulated as follows:

$$R_0 = \frac{VCb_h}{r_h}$$

We accounted for global spatiotemporal dynamics of the mosquito populations. The spatial resolution of the global models was 0.5 × 0.5 deg. We used daily time-steps for solving the models, and presented the results at monthly time-steps. We assume well-mixed mosquito populations within 0.5 × 0.5 deg grid-cells. The models compute the population-density of mosquitoes independently in each grid cell. Accordingly, we recover the spatiotemporal pattern in population-density of *Ae. aegypti* and *Ae. albopictus*, respectively, accounting for the effects of the environment, in terms of temperature, precipitation, and day-light length, on mosquito-population density. The dynamical systems were, respectively, numerically solved using the `deSolve` package[11] in R for the population-density of blood-feeding adult mosquitoes.

The results on the length of transmission-season (LTS) and the population at risk (PAR) were both based on R_0 values. The ratio between number of mosquitoes that could potentially be in contact with humans, to the number of humans, is central to the R_0 value. The original mosquito-population models provide results in terms of the number of individuals of *Ae. aegypti* per breeding site (X), or the number of *Ae. albopictus* per hectare (Y).[8,9] In order to appropriately consolidate mosquito population density and human population density (p), we multiplied X by $f(p,a,c) = a * g(p,c)$ where a equals to the number of breeding-sites per human, and Y by $f(p,a/b,c) = a * g(p,c)/b$ where b equals the average number of breeding sites per hectare. The function $g(p,c) = p^2/(c^2 + p^2)$ is an increasing sigmoidal function that equals the viability of domesticated mosquito-populations in relation to human population density. Accordingly, $f(p,a,c)$ is a multiplicative factor to R_0, which allowed us to straightforwardly estimate correct values for a, a/b and c by fitting R_0 to R_0-data that was available for a subset of the spatiotemporal points.[12]

Technical details of the statistical dengue model

Let $Y_{i,t}$ be the number of dengue cases for administrative unit $i = 1, \cdots, I$ at month $t = 1, \cdots, T$ where I is the total number of administrative units in the data set, and T the total number of time steps for which the model will be run be modelled using a generalised additive mixed model (GAMM). Models were fitted using a negative binomial specification to account for potential over-dispersion in the data. The general algebraic definition of the models is a simplified version of a model previously used [13] and is given by:

$$\log(\mu_{i,t}) = \alpha + \log(P_{i,a[t]}) + \sum_{k=1}^{K} f(X_{i,t,k}) + \epsilon_j D_{a[t],j} + \gamma_{i,a[t]} + \delta_{m[t]} + u_i$$

where $E[Y] \equiv \mu$ and $var(Y) \equiv \mu(1+\mu/\varphi)$; φ is the dispersion parameter; α denotes the intercept; $\log(P_{i,a[t]})$ indicates the logarithm of the total population for each administrative unit i and year $a[t]$, included as an offset; X is a matrix of $k = 1, \cdots, 3$ smooth functions of the meteorological variables (mean temperature, precipitation and relative humidity) defined as linear regression splines f; D is the population density per km^2 in administrative unit i at year $a[t]$ with regression coefficient ϵ. Long-term trends are modelled using unstructured random effects for each year ($\gamma_{i,a[t]}$). Seasonal trends are accounted for using cyclic cubic splines δ for each calendar month $m[t]$ so that the first month of the year depends on the last month of the previous year.[14] Unknown confounding factors, such as public health interventions were incorporated using unstructured random effects (u_i) for each administrative unit. Models were fitted in R version 3.6.1 using the `mgcv` package.[14]

Technical details of the VECTRI model

The mathematical model of VECTRI is outlined in Tompkins and Ermert (20013).[15] In each grid cell of the simulation domain, it uses a daily timestep to integrate a set of prognostic equations for the larvae density divided into a number of growth-stage bins, the vector density divided into sporogonic status bins and the progression of the disease in a human host using a classical compartmental SEIR (susceptible, exposed, infectious, recovered) approach, with the exposed category also divided into disease-progression bins, and the immune R class divided between infectious and non-infectious states. Temperature impacts the larvae, adult vector and parasite development rates and the vector and larvae mortality. The model version used is v1.8.2. Differences to the original model v1.2.6[15] include: (i) an improved hydrological treatment of puddles;[16] (ii) the replacement of the bin-resolved gonotrophic cycle in adult mosquitoes with a standard formulation using a single prognostic equation. This change has little impact on results but benefits greatly the speed of execution and memory requirements; (iii) the implementation of host immunity based on Asare et al., (2016);[17] (iv) an adjusted temperature used in the sporogonic cycle to account for the indoor hut temperature for a proportion of the day;[18] (v) diffusion of mosquitoes between adjacent model grid cells;[19] and (vi) a machine learning calibration technique outlined above and detailed in Tomkins and Thompson (2018).[4]

Supplementary Figures

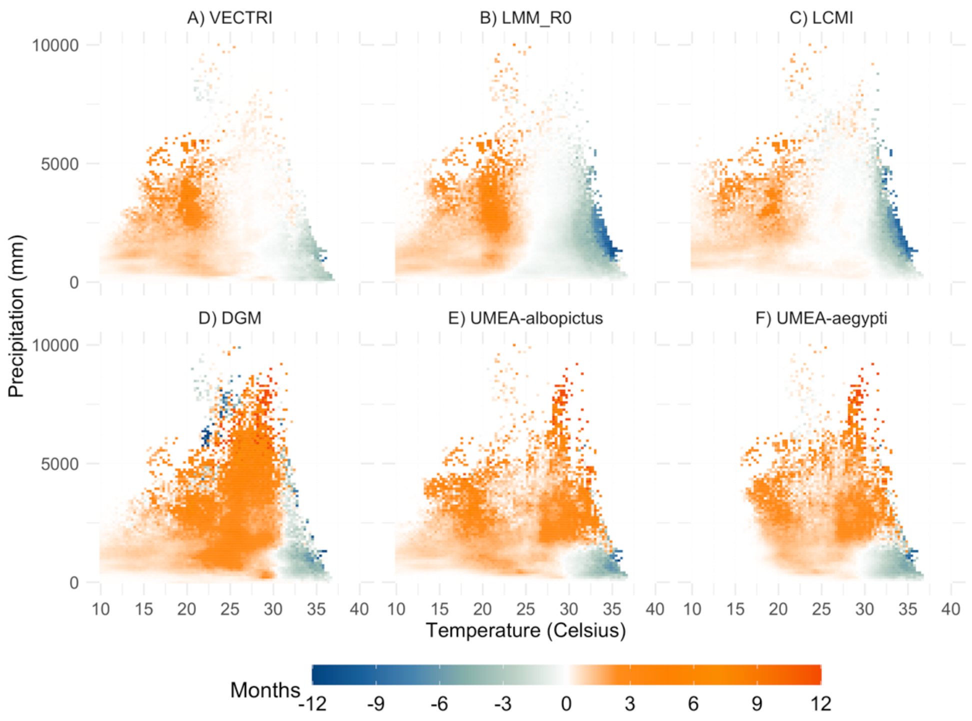

Fig A1: Average future change in LTS (months) as a function of average annual temperature (*x*-axis in degrees Celsius) and rainfall (*y*-axis in mm year^{-1}) for A) VECTRI, B) LMM_R$_0$, C) LCMI, D) DGM, E) UMEÅ-*albopictus*, and F) UMEÅ-*aegypti*. Differences in LTS are calculated between future time slices (2010–2039, 2040–2069 and 2070–2099) and the 1970–1999 reference period for the historical simulations. Future time slices are calculated for all RCP-SSP2 simulations and all grid points per disease model. Associated average temperature and rainfall values are calculated for the same future time slices.

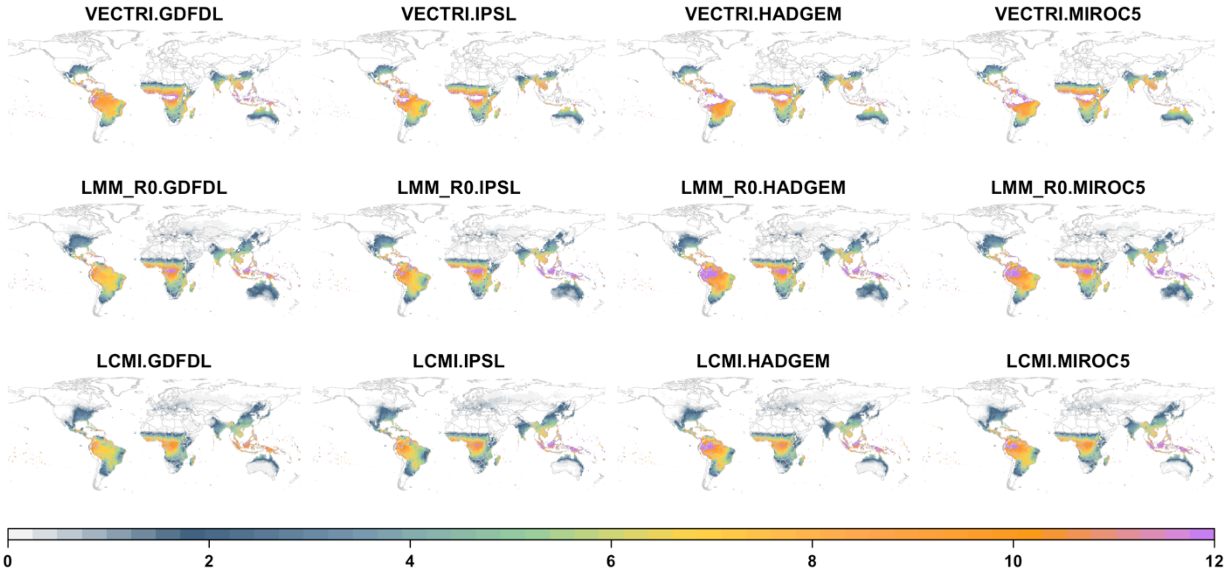

Fig A2: Ensemble mean of the simulated length of the transmission season of malaria (in months) over the baseline period 1970–1999 stratified per climate model.

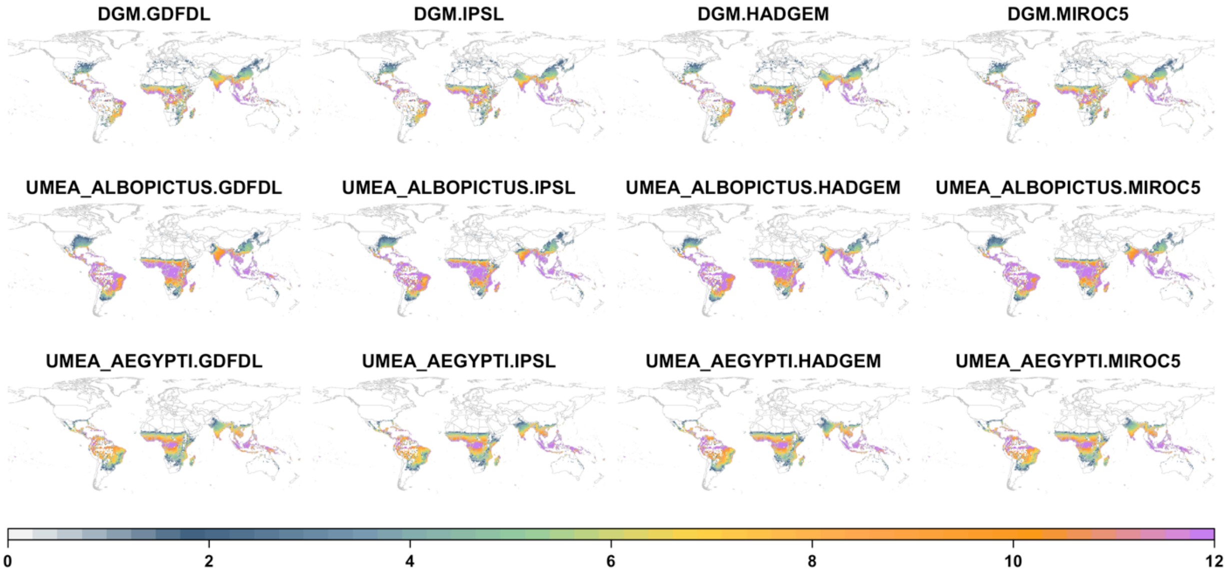

Fig A3: Ensemble mean of the simulated length of the transmission season of dengue (in months) over the baseline period 1970–1999 stratified per climate model.

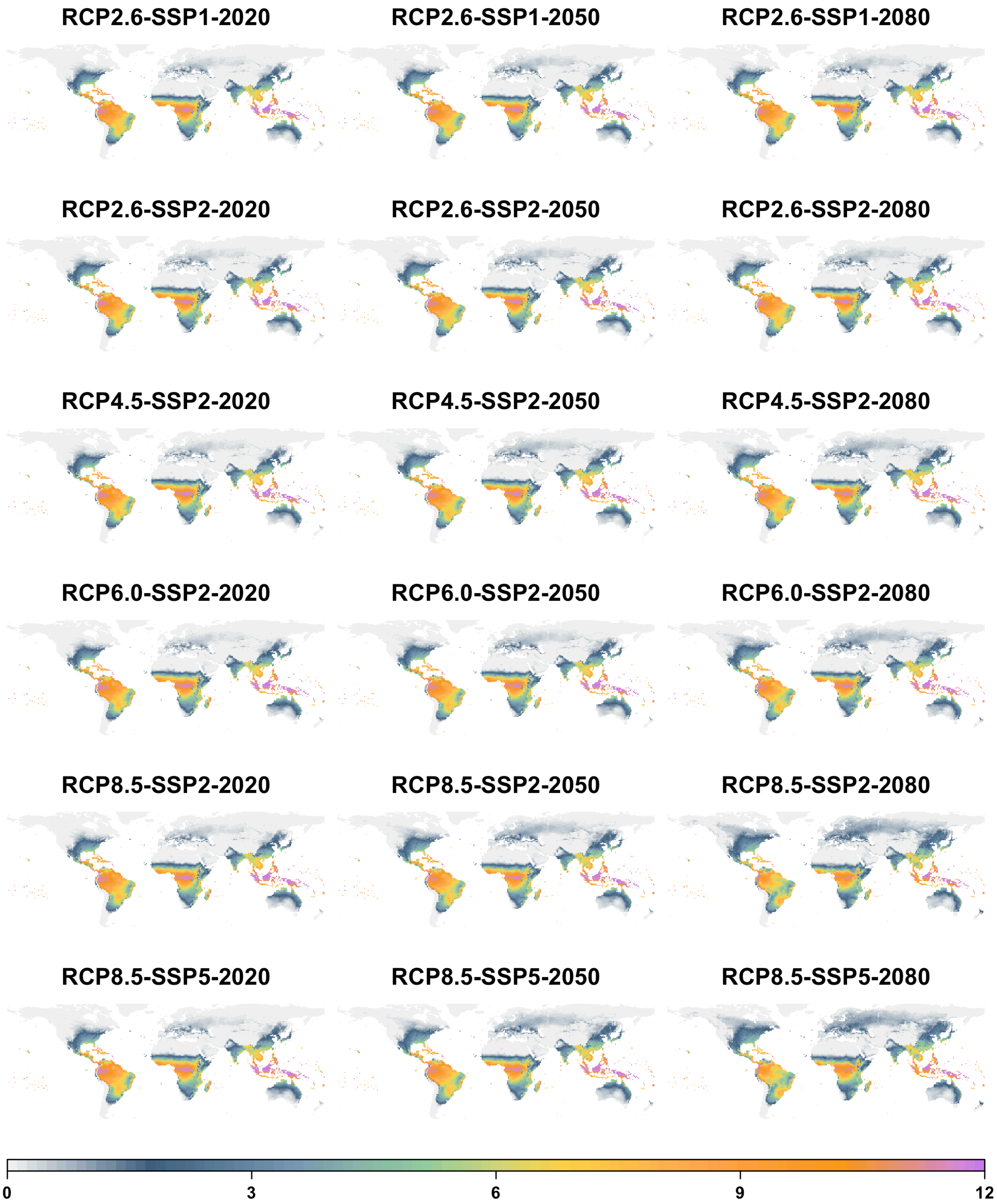

Fig A4: Ensemble mean of the simulated length of the transmission season of malaria over the period 2070–2099 stratified per RCP-SSP scenario.

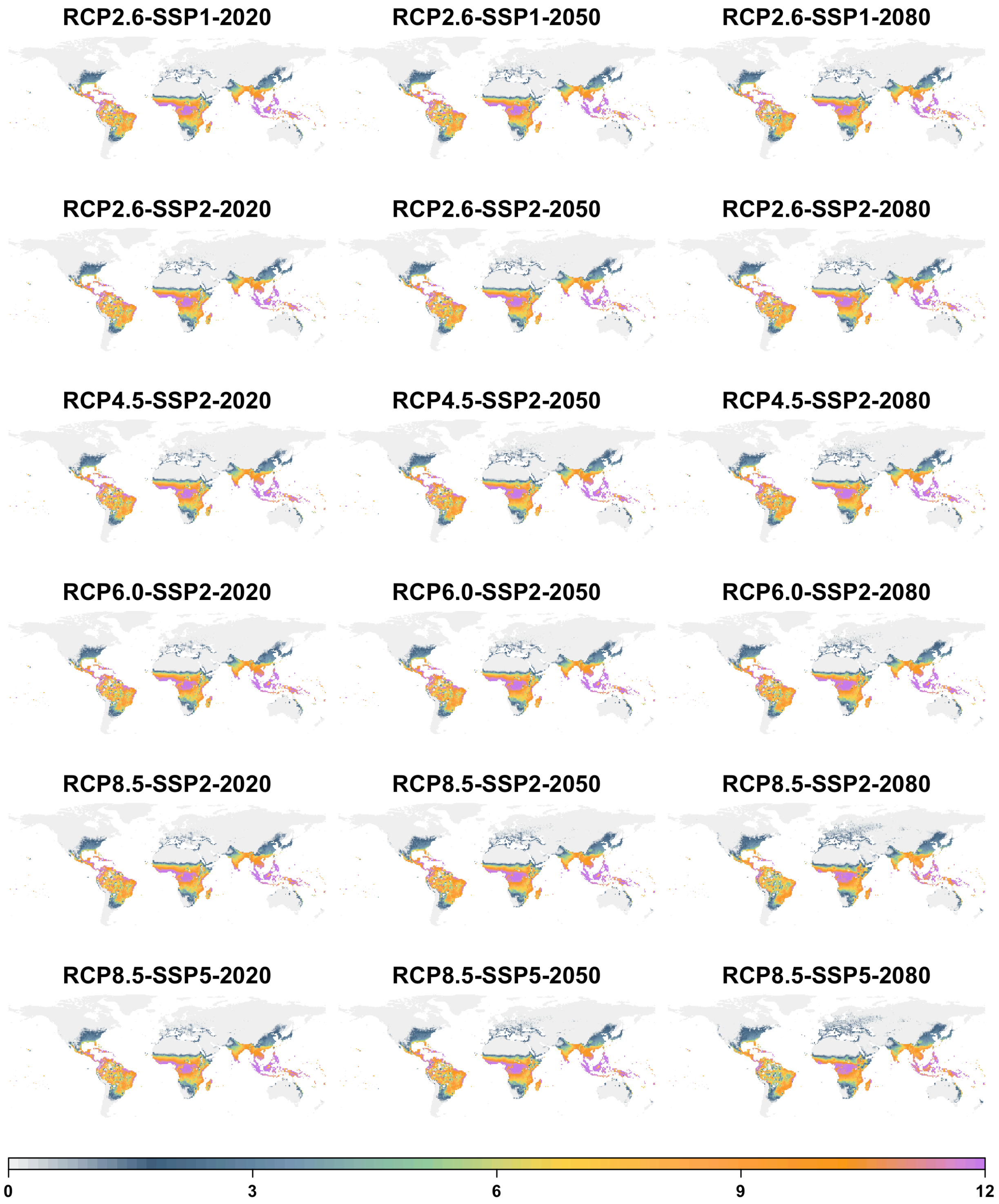

Fig A5: Ensemble mean of the simulated length of the transmission season of dengue over the period 2070–2099 stratified per RCP-SSP scenario.

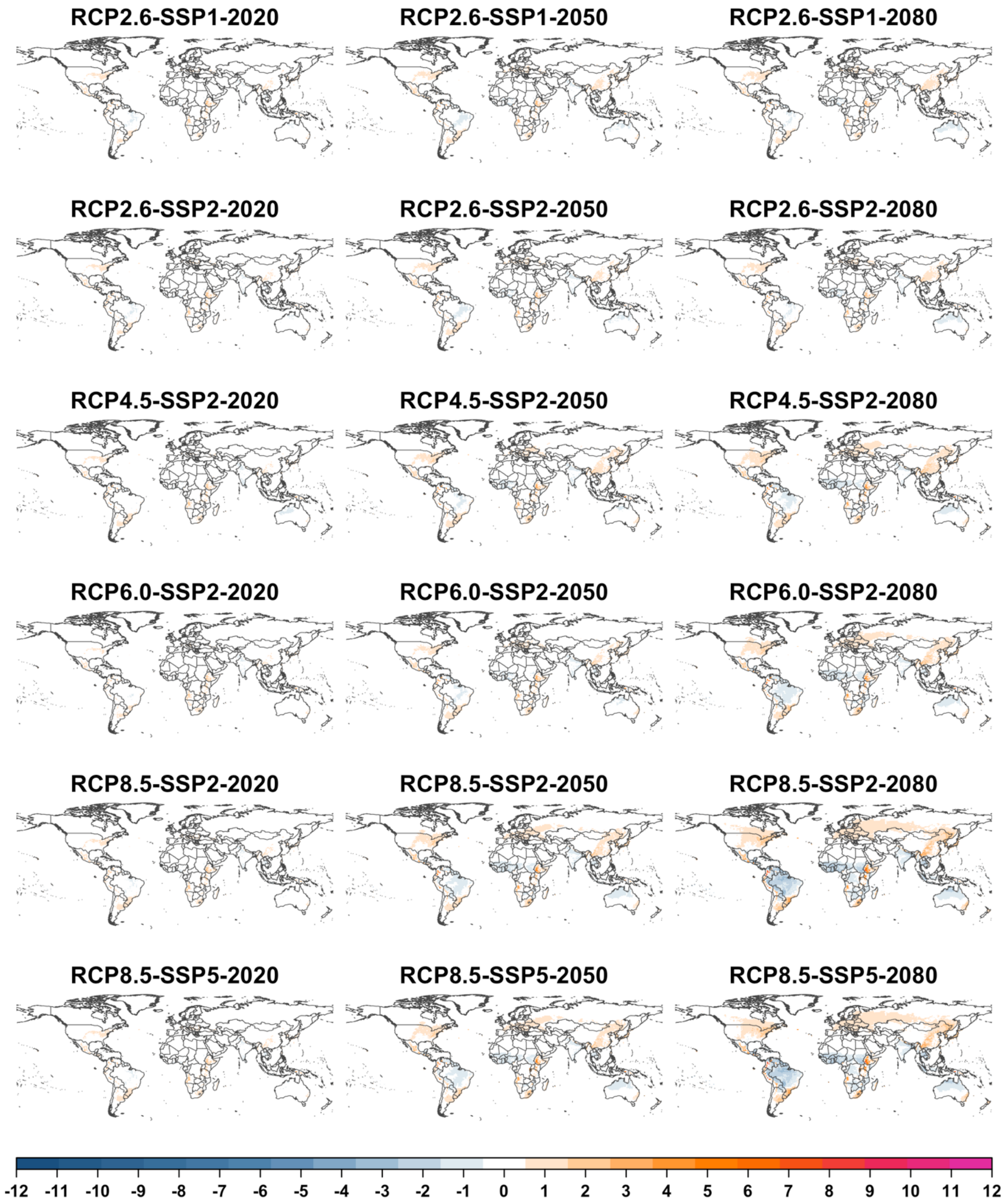

Fig A6: Ensemble mean of the simulated changes in the length of the transmission season of malaria over the period 2070–2099 stratified per RCP-SSP scenario.

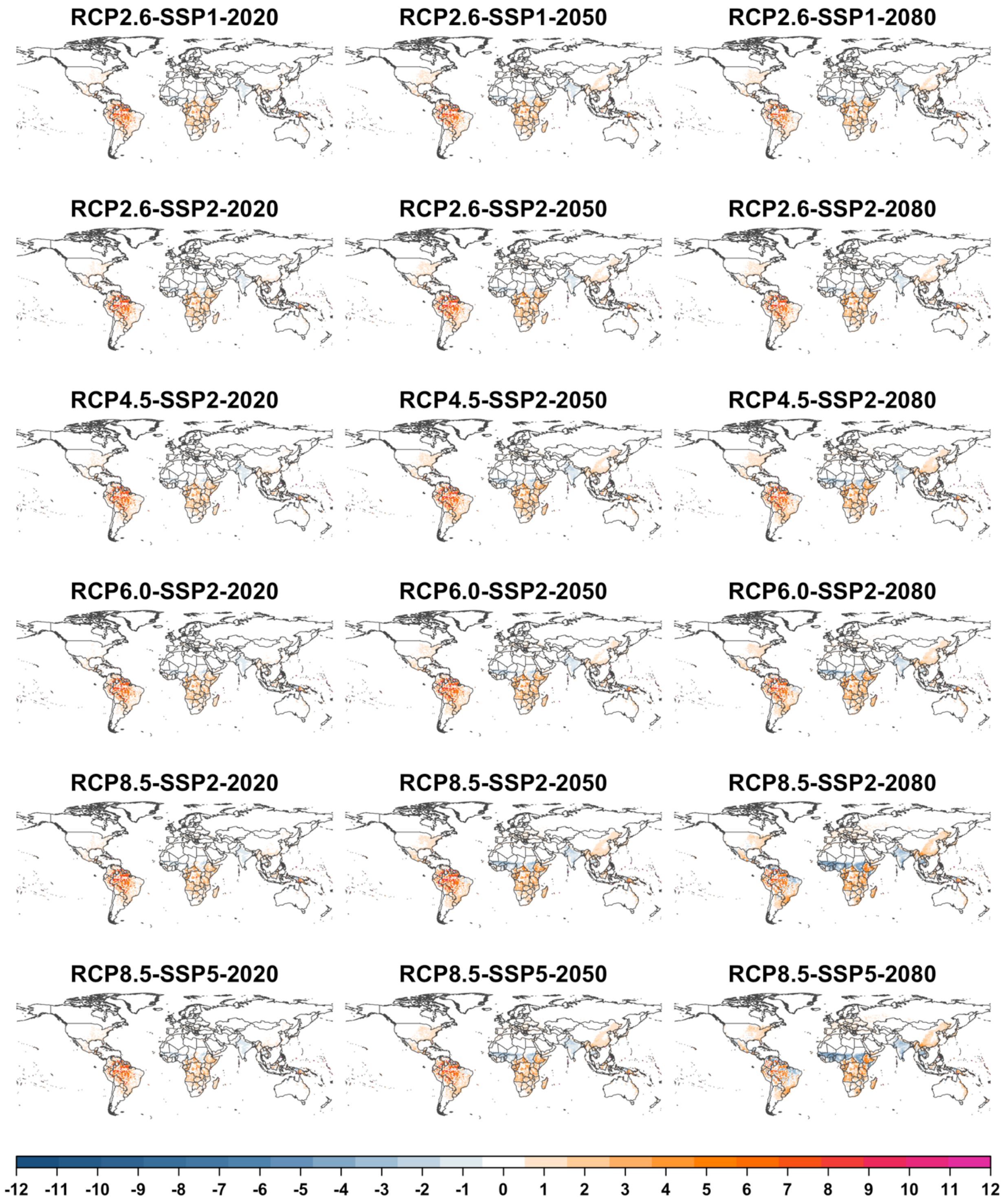

Fig A7: Ensemble mean of the simulated changes in the length of the transmission season of dengue over the period 2070–2099 stratified per RCP-SSP scenario.

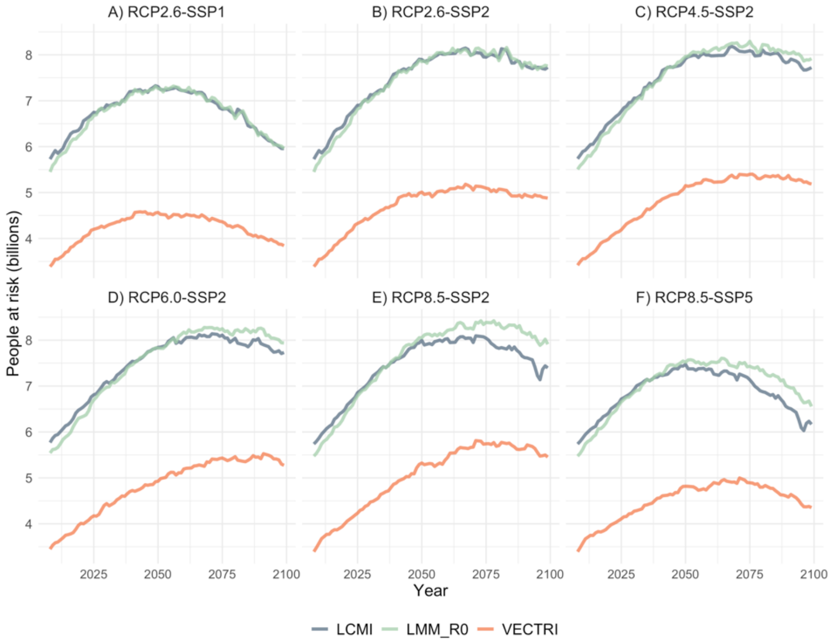

Fig A8: Ensemble mean of the simulated changes in the population at risk of malaria transmission per RCP-SSP scenario.

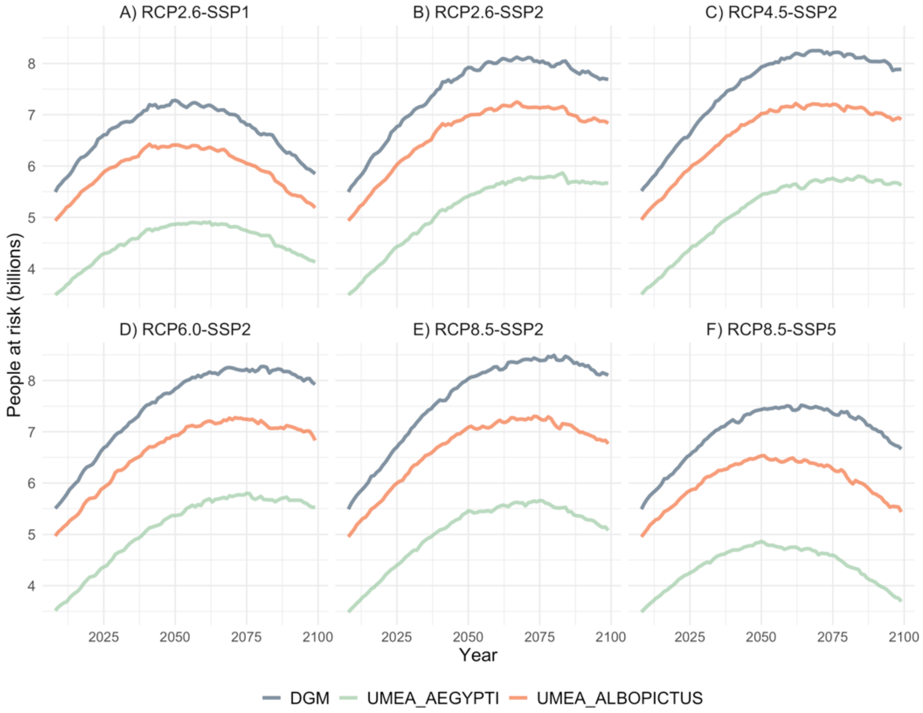

Fig A9: Ensemble mean of the simulated changes in the population at risk of dengue transmission per RCP-SSP scenario.

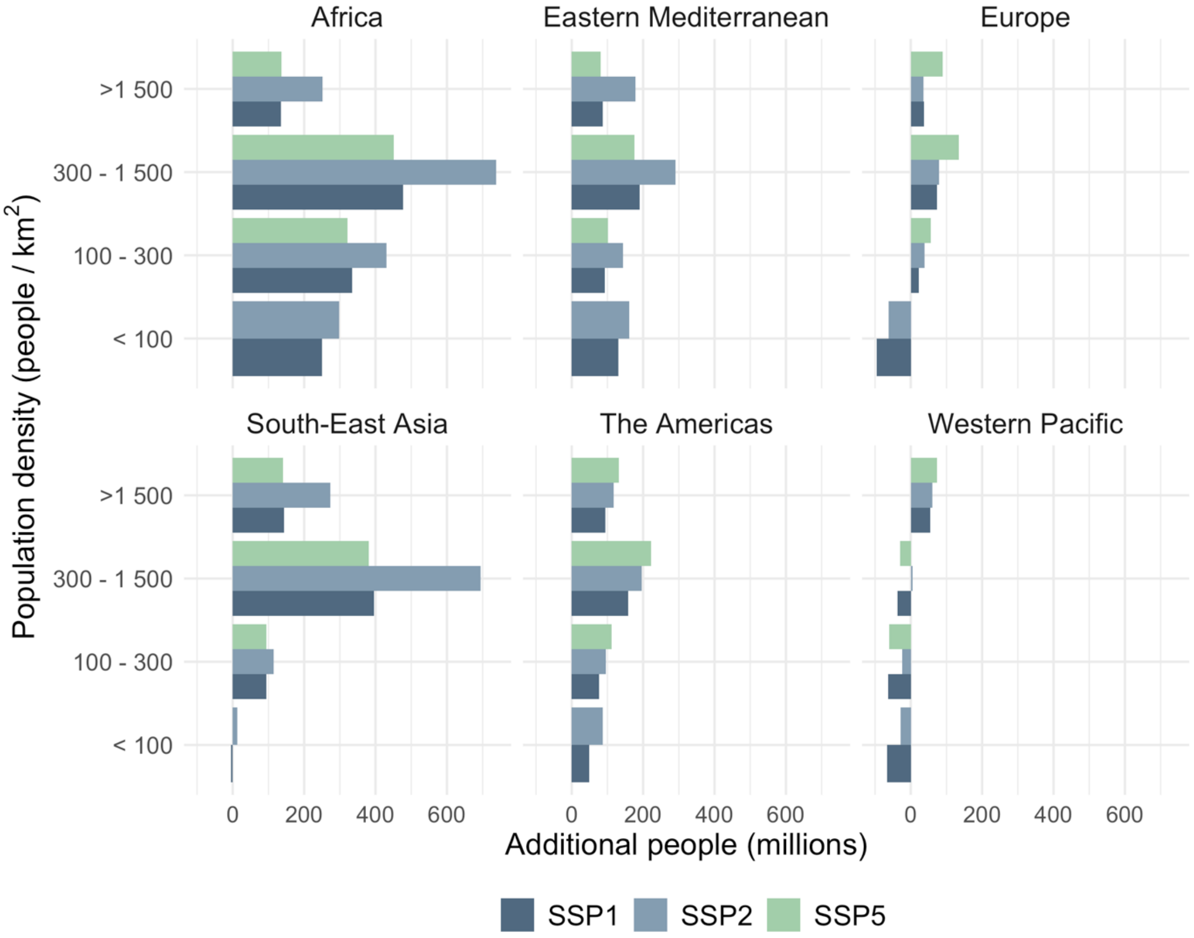

Fig A10: Mean projected population density per SSP scenario for the period 2070–2099.

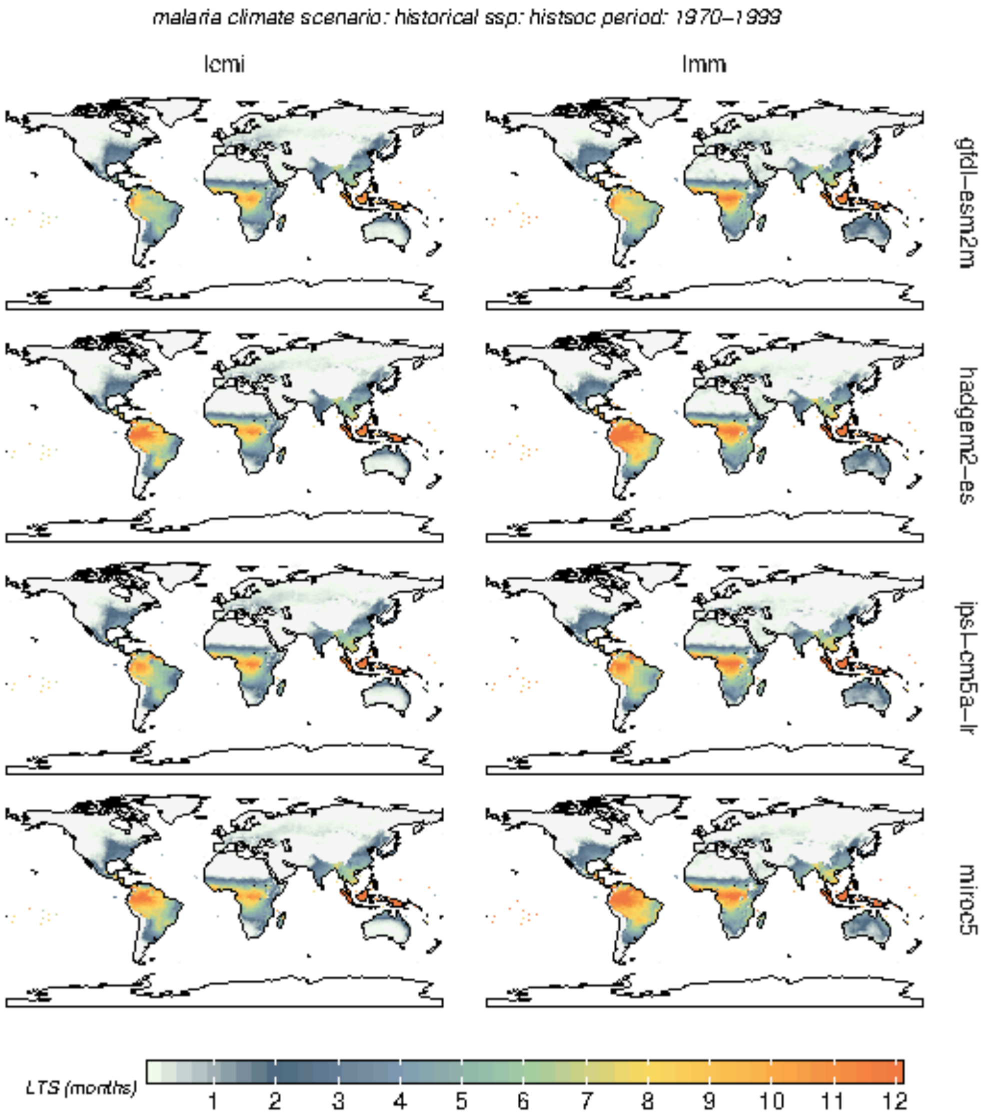

Fig A11: Simulated length of the transmission season of malaria for the period 1970–1999 for the LCMI and LMM_R_0 models stratified by climate model.

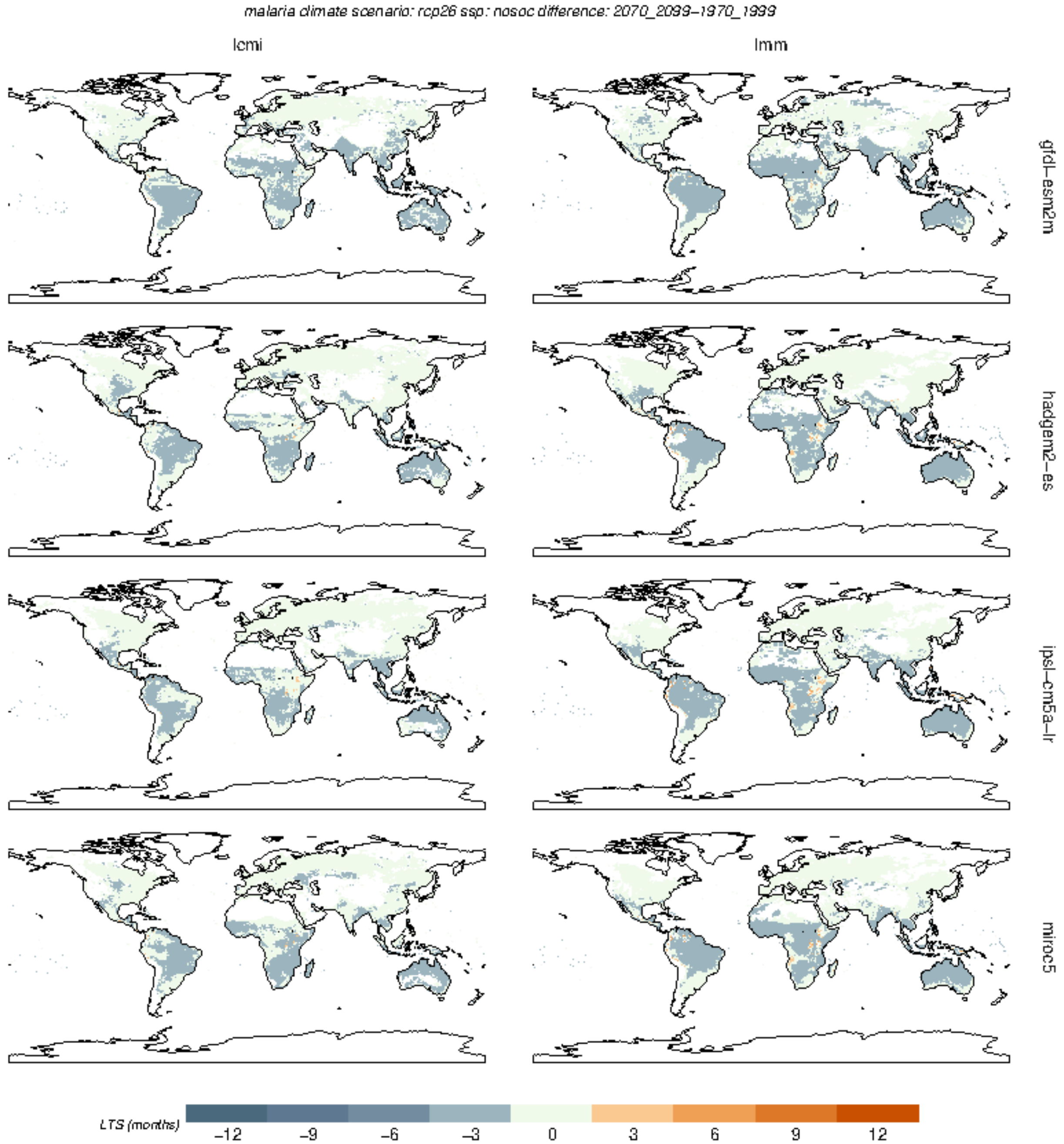

Fig A12: Simulated changes in the length of the transmission season of malaria over the period 2070–2099 for the LCMI and LMM_R_0 models, and for the RCP2.6 scenario stratified by climate model.

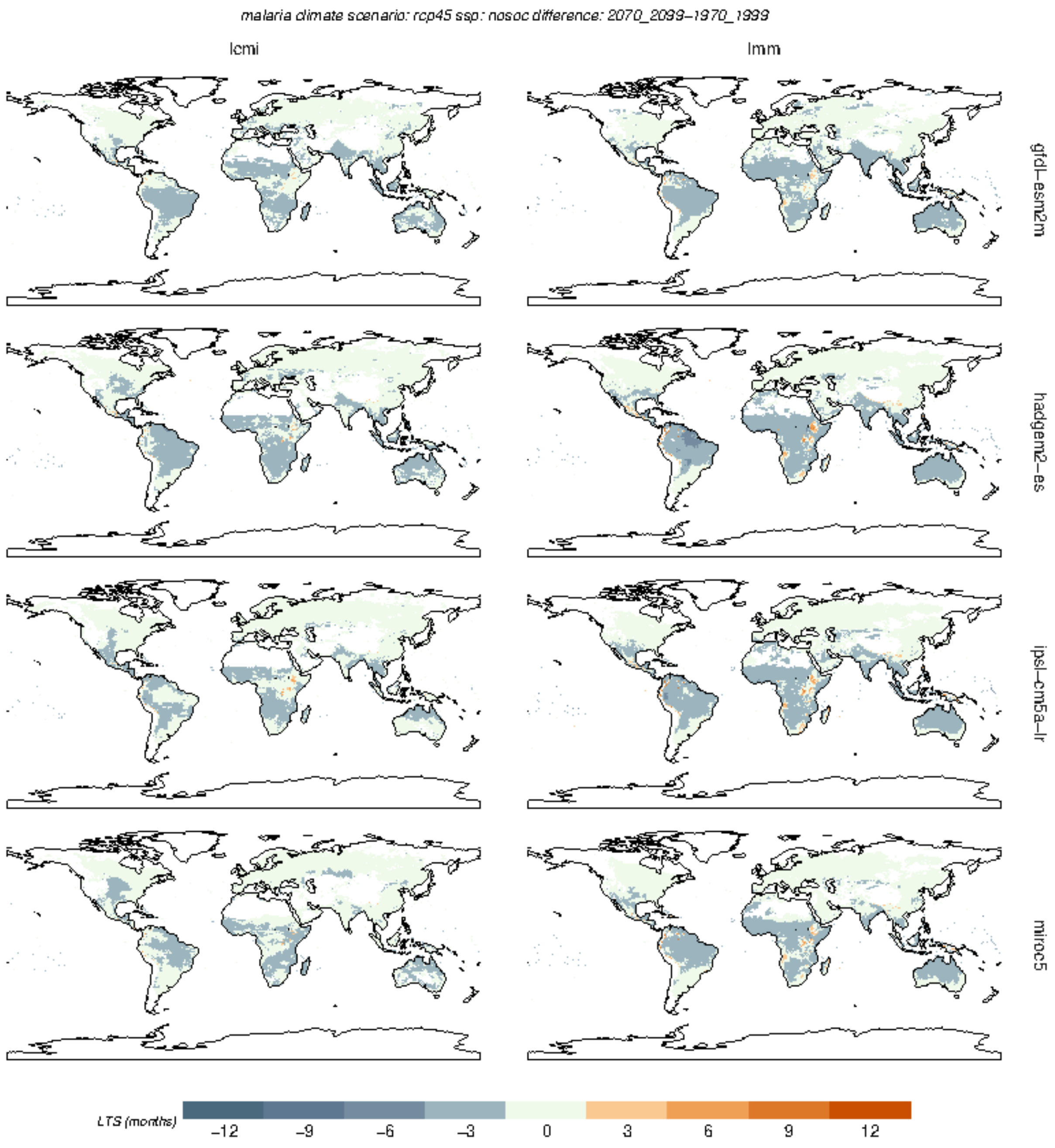

Fig A13: Simulated changes in the length of the transmission season of malaria over the period 2070–2099 for the LCMI and LMM_R_0 models, and for the RCP4.5 scenario stratified by climate model.

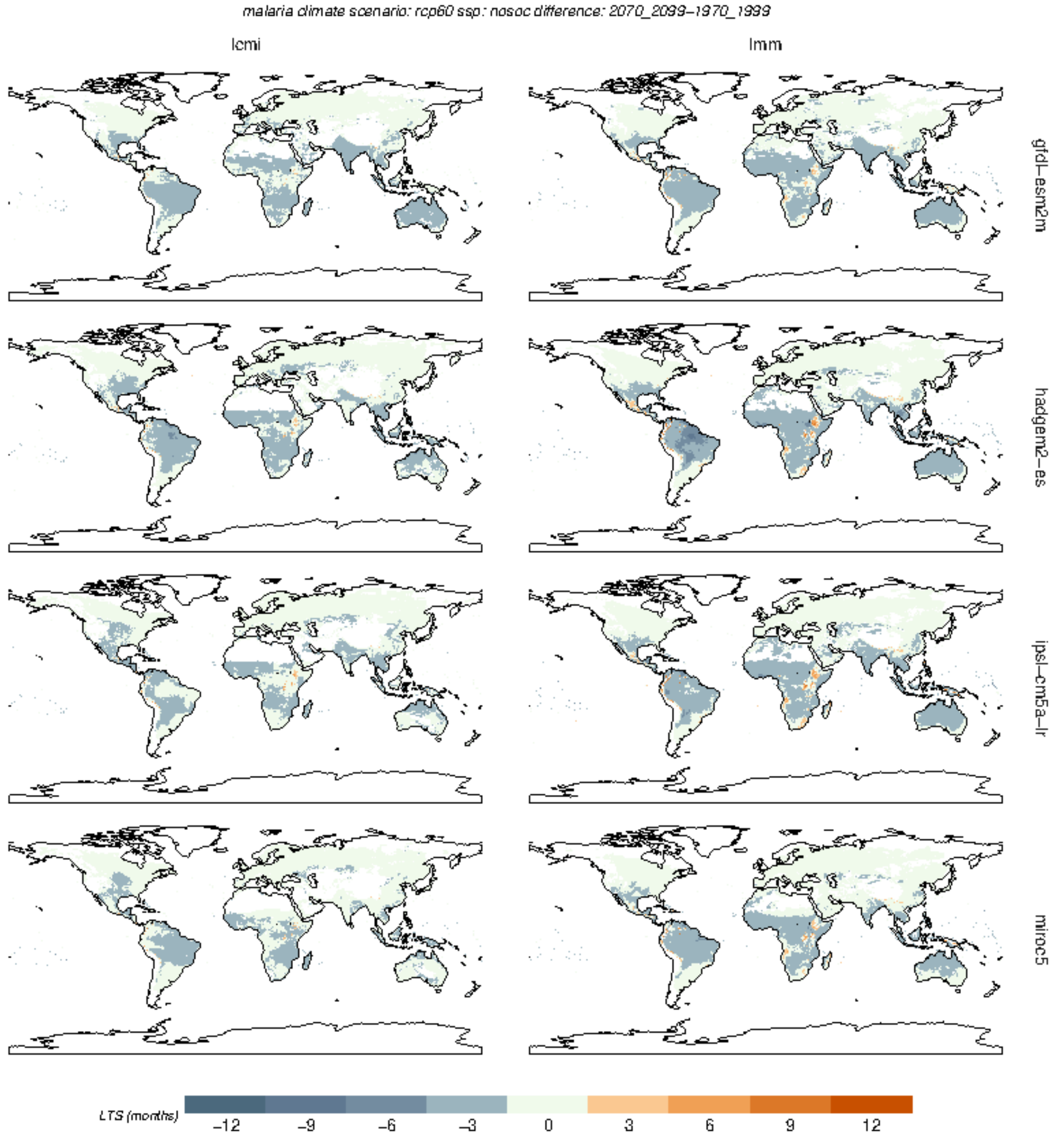

Fig A14: Simulated changes in the length of the transmission season of malaria over the period 2070–2099 for the LCMI and LMM_R_0 models, and for the RCP6.0 scenario stratified by climate model.

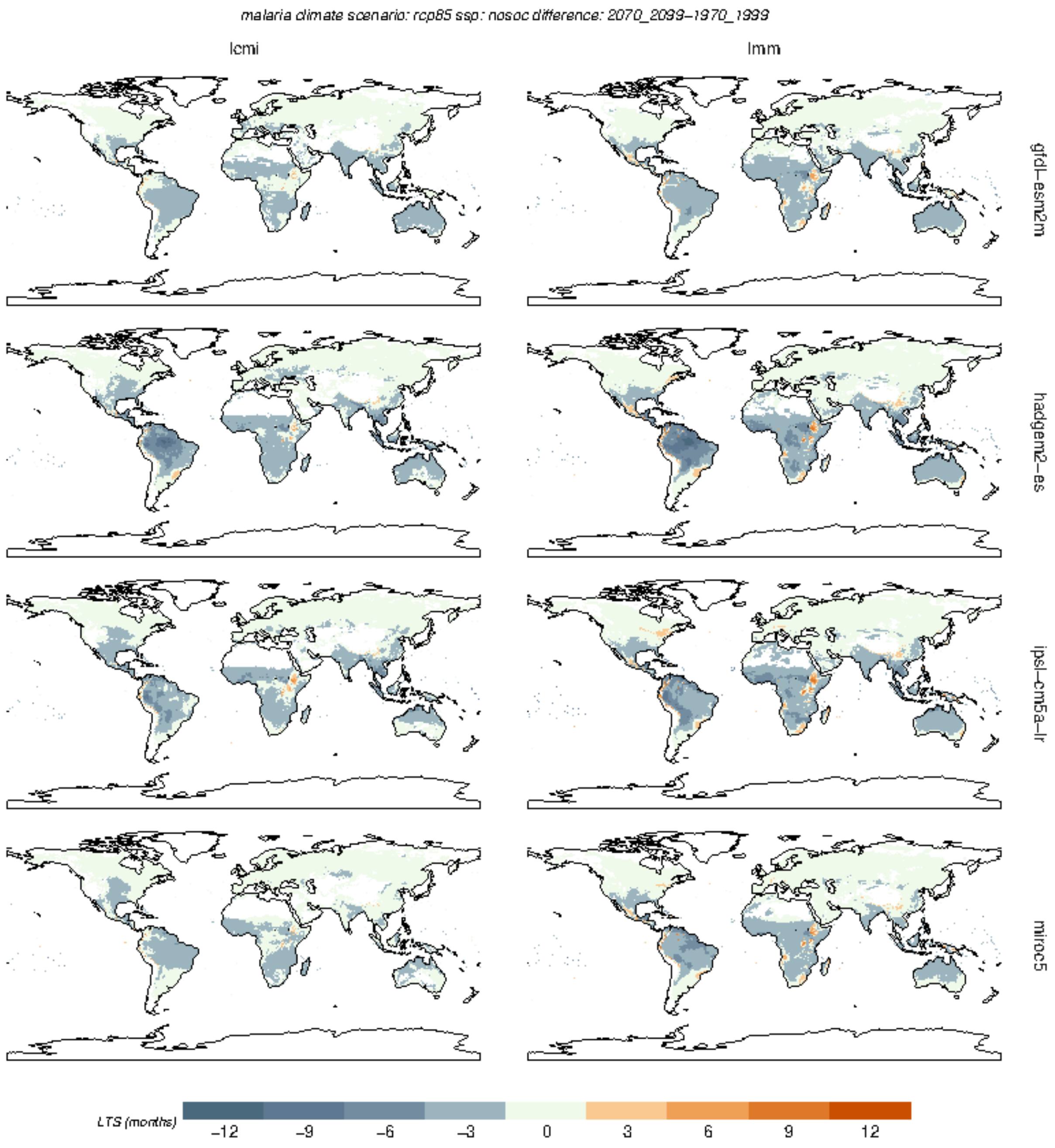

Fig A15: Simulated changes in the length of the transmission season of malaria over the period 2070–2099 for the LCMI and LMM_R_0 models, and for the RCP8.5 scenario stratified by climate model.

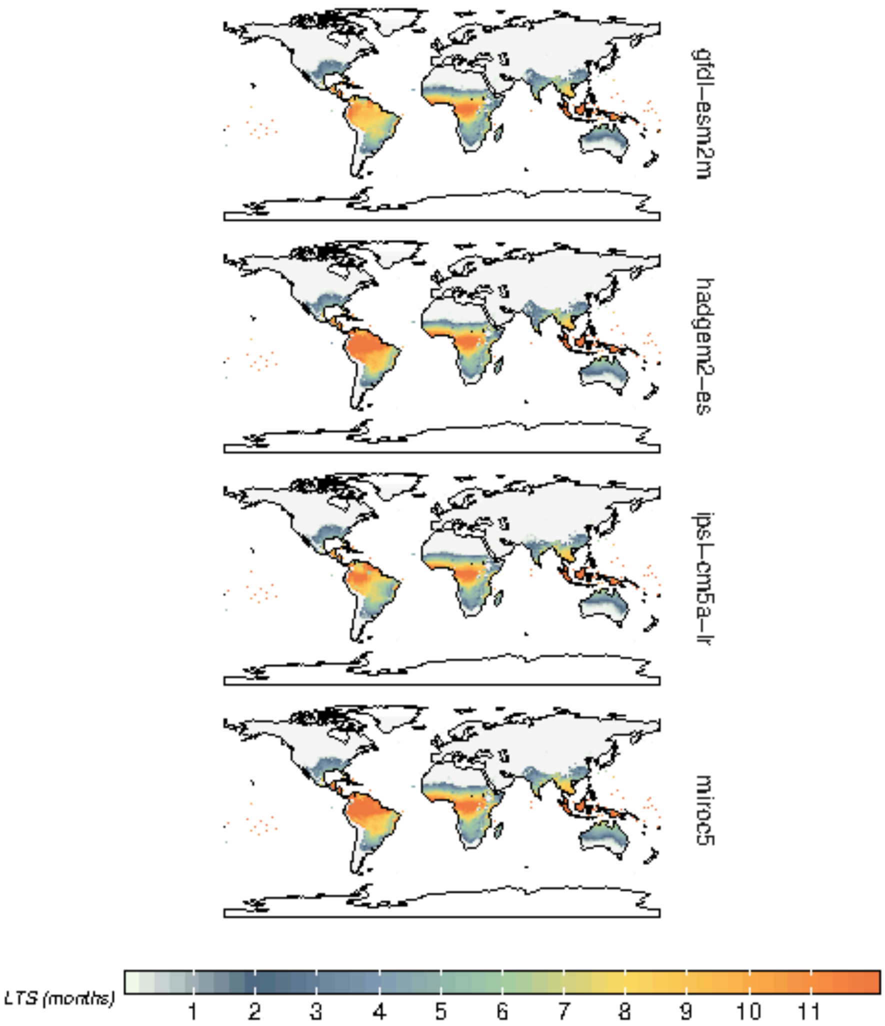

Fig A16: Simulated length of the transmission season of malaria over the period 1970–1999 for the VECTRI model stratified by climate model.

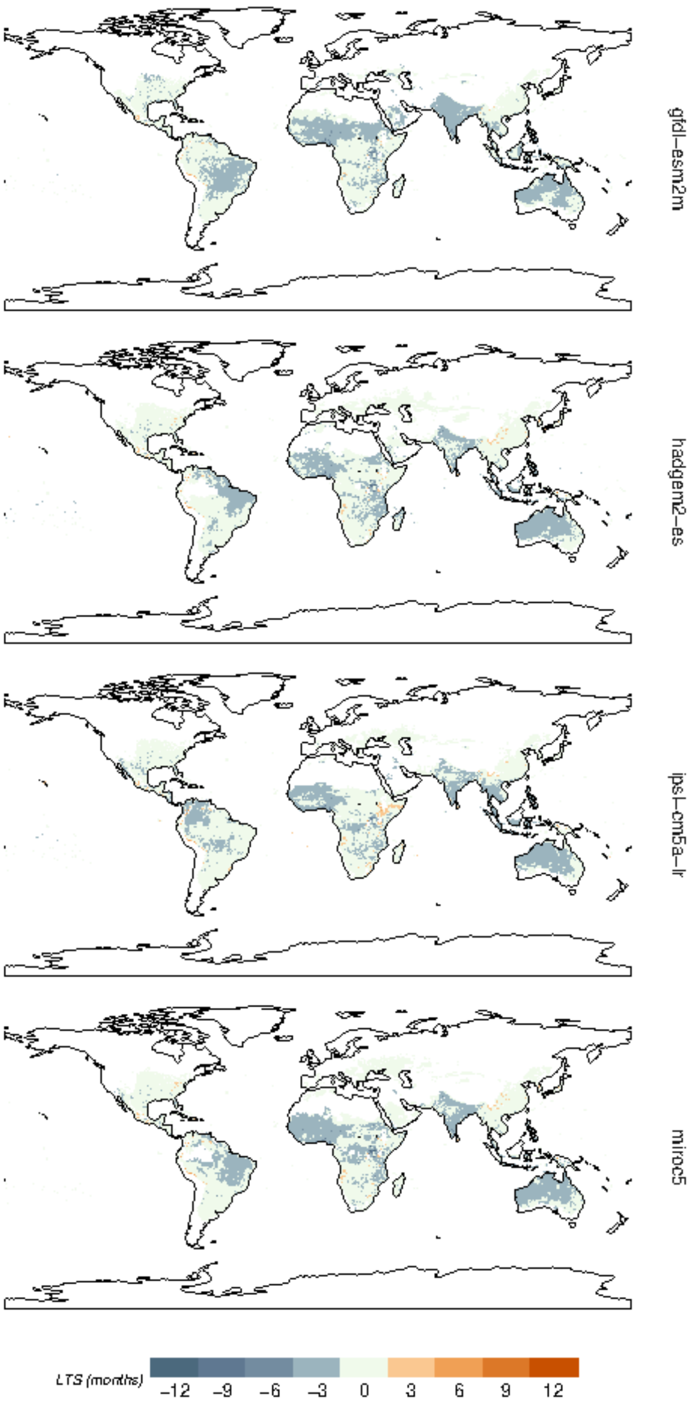

Fig A17: Simulated changes in the length of the transmission season of malaria over the period 2070–2099 for the VECTRI model, and for the RCP2.6-SSP1 scenario stratified by climate model.

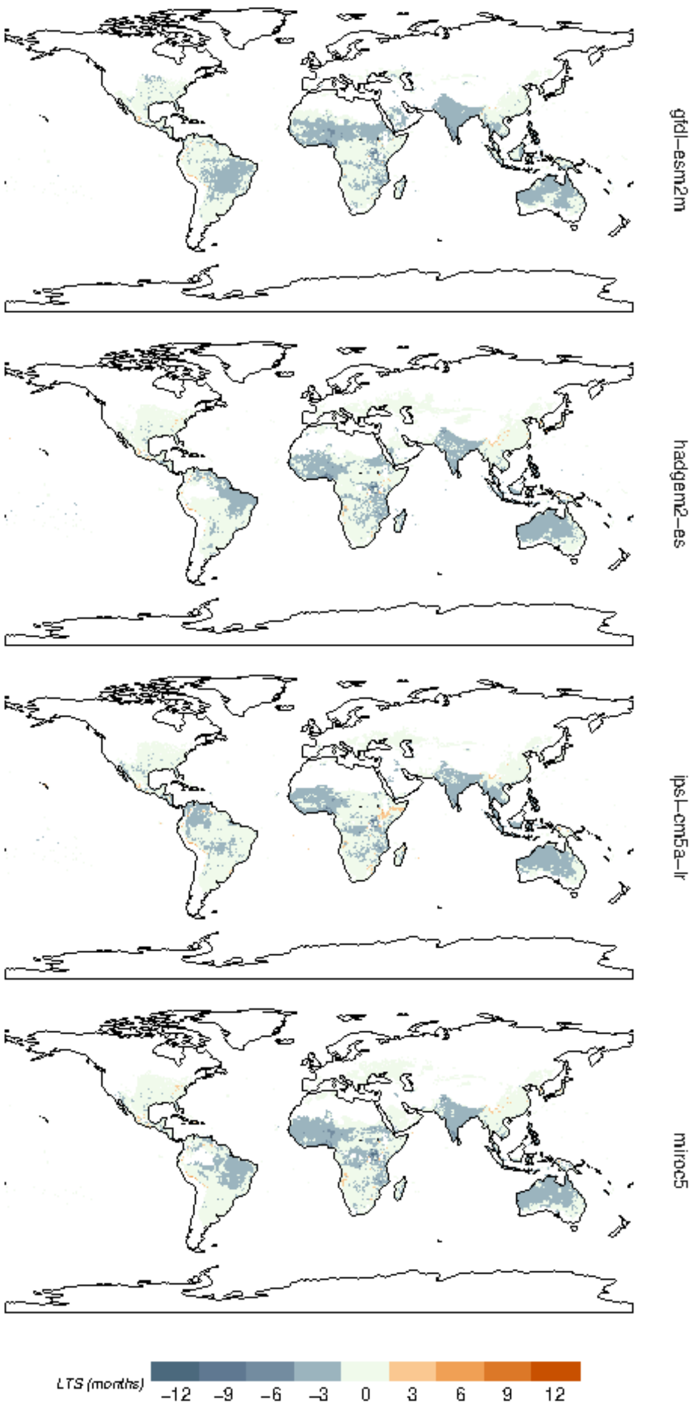

Fig A18: Simulated changes in the length of the transmission season of malaria over the period 2070–2099 for the VECTRI model, and for the RCP2.6-SSP2 scenario stratified by climate model.

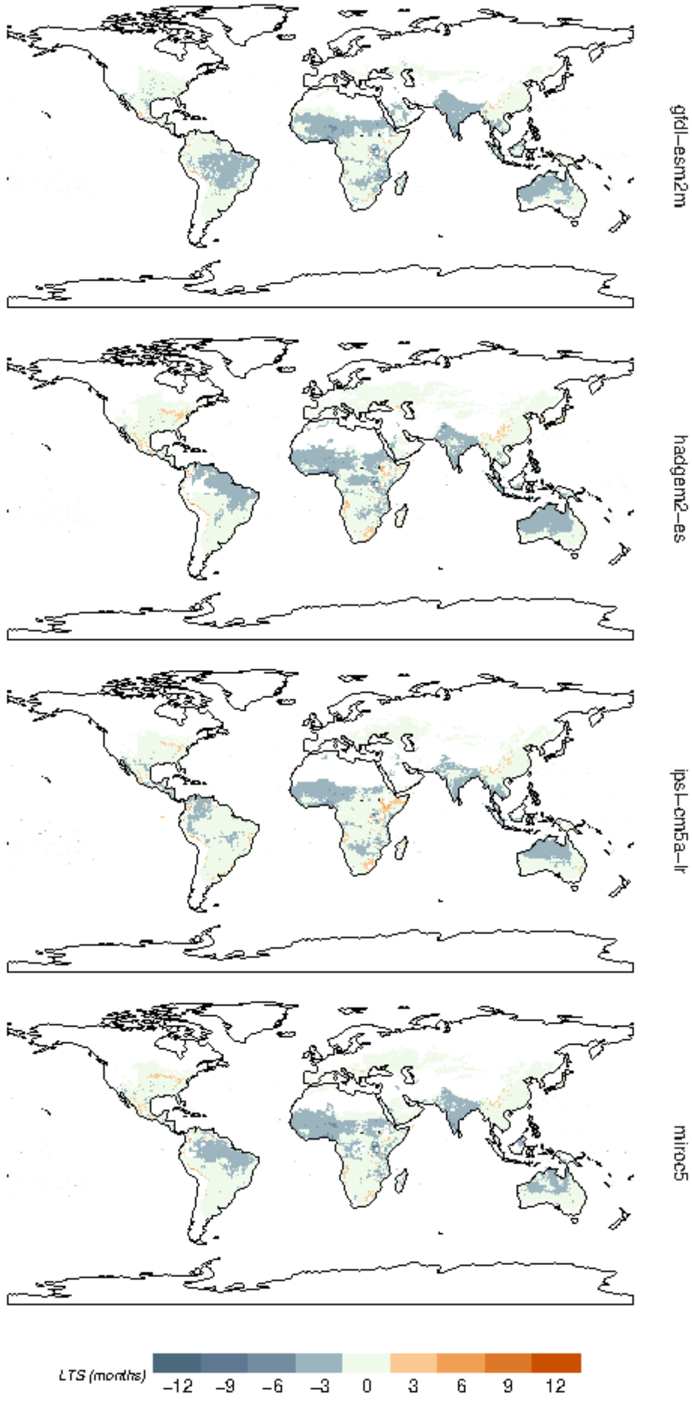

Fig A19: Simulated changes in the length of the transmission season of malaria over the period 2070–2099 for the VECTRI model, and for the RCP4.5-SSP2 scenario stratified by climate model.

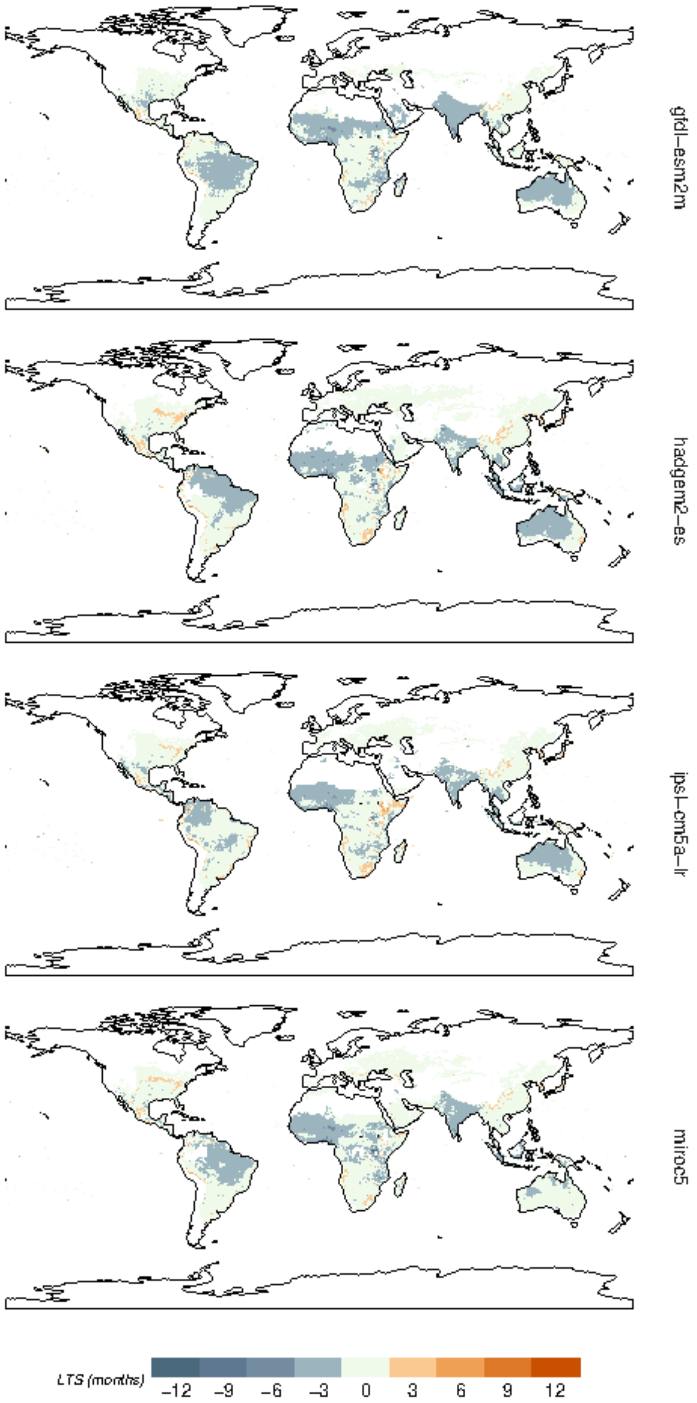

Fig A20: Simulated changes in the length of the transmission season of malaria over the period 2070–2099 for the VECTRI model, and for the RCP6.0-SSP2 scenario stratified by climate model.

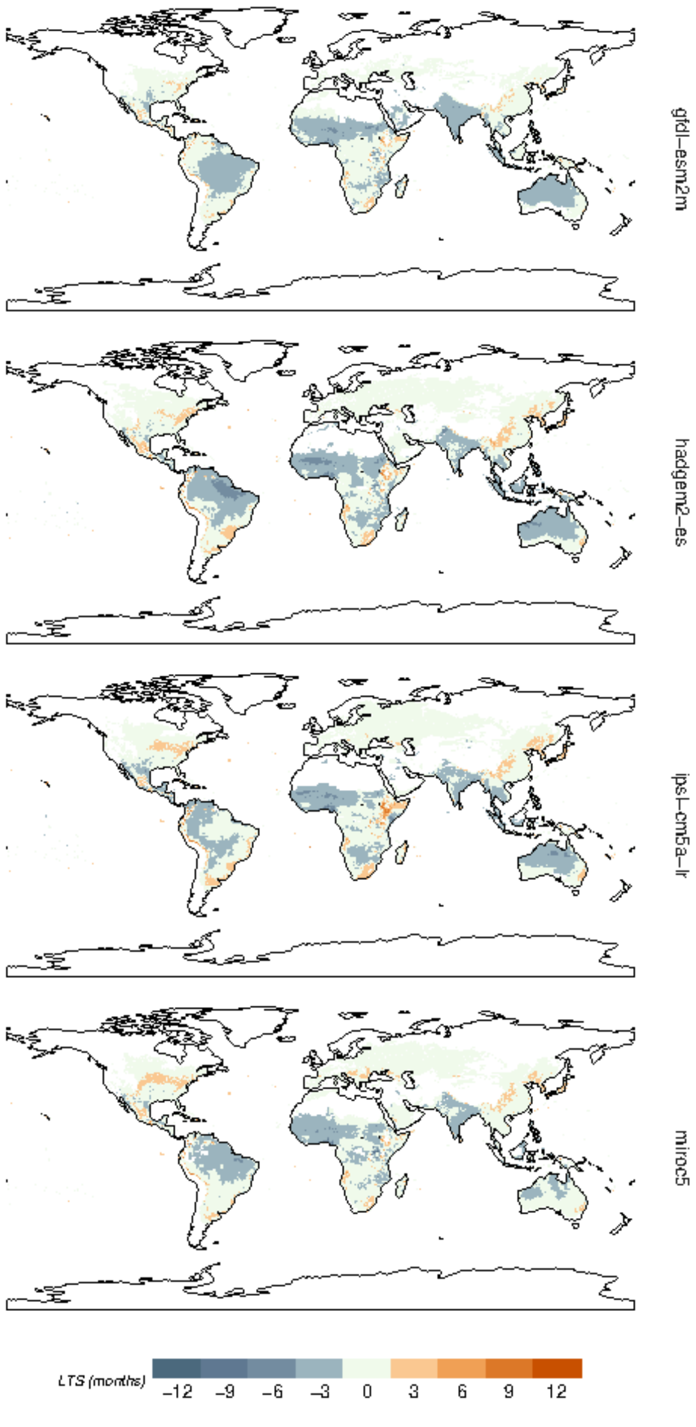

Fig A21: Simulated changes in the length of the transmission season of malaria over the period 2070–2099 for the VECTRI model, and for the RCP8.5-SSP2 scenario stratified by climate model.

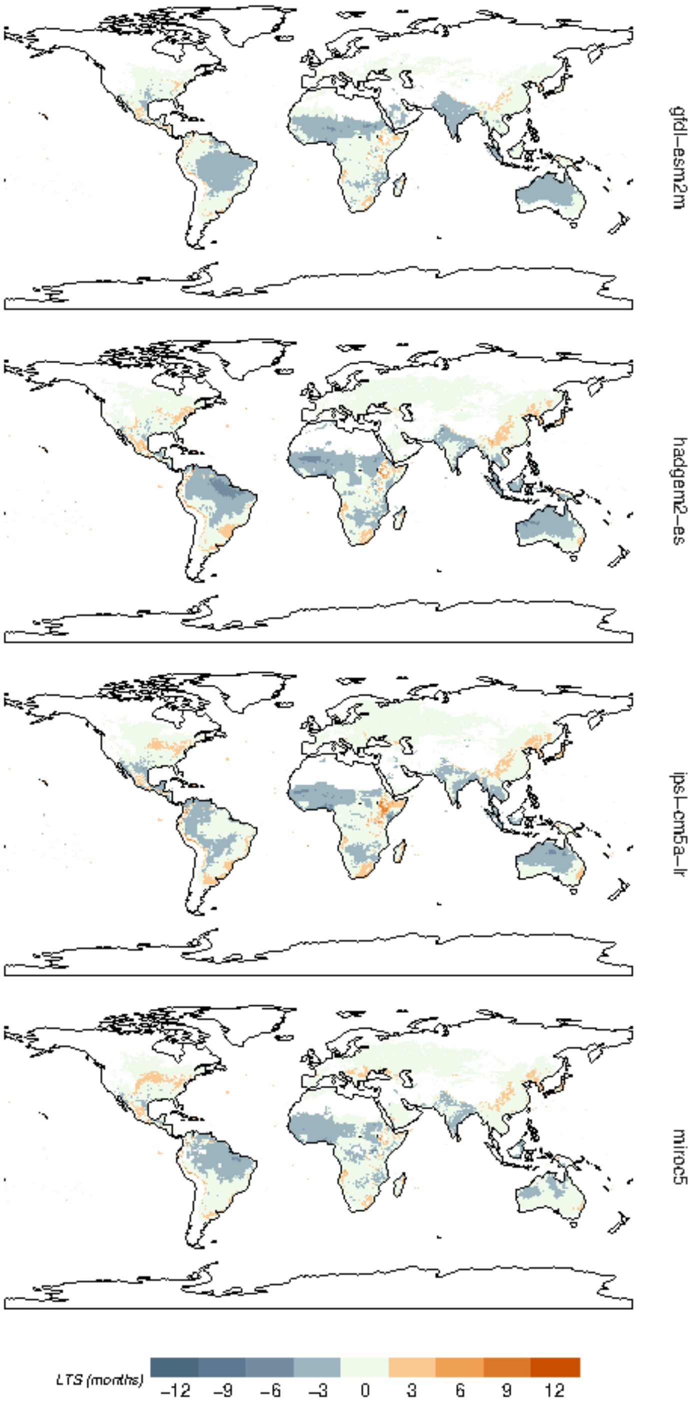

Fig 22: Simulated changes in the length of the transmission season of malaria over the period 2070–2099 for the VECTRI model, and for the RCP8.5-SSP5 scenario stratified by climate model.

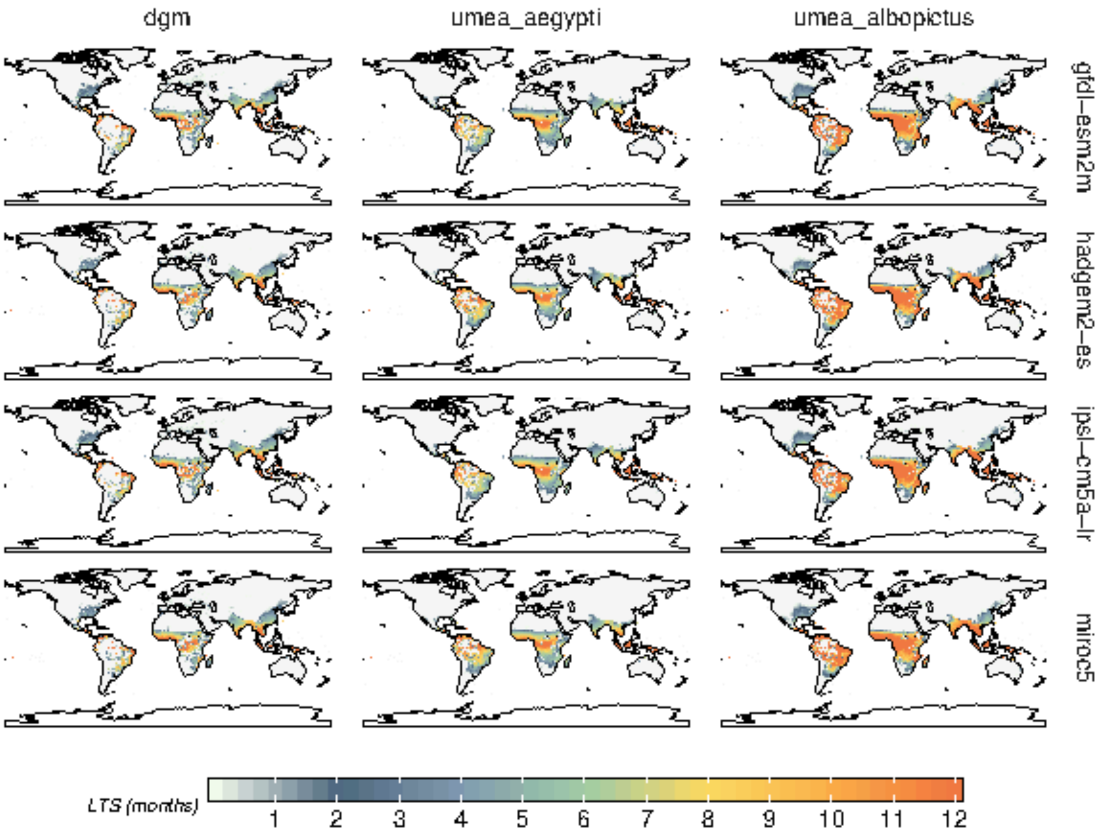

Fig A23: Simulated length of the transmission season for dengue over the period 1970–1999 stratified by climate model.

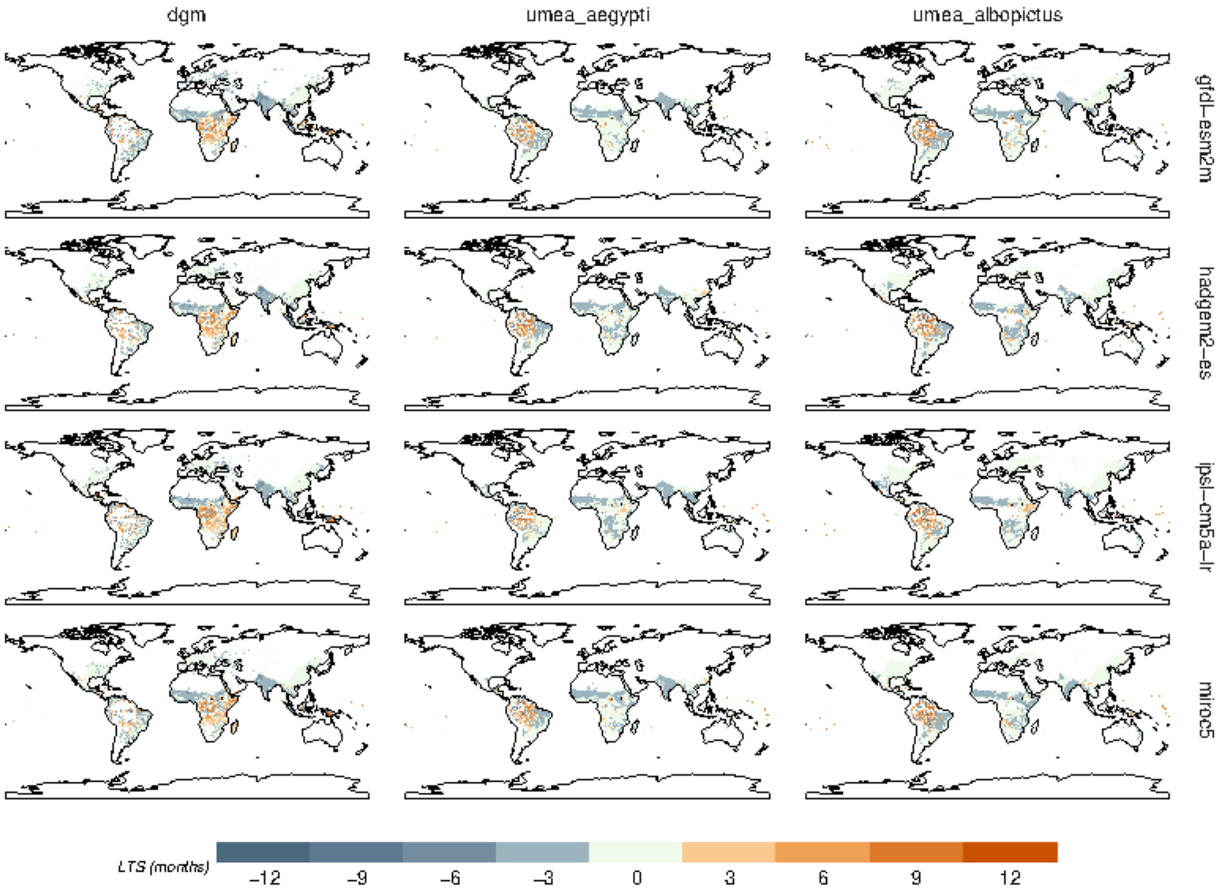

Fig A24: Simulated changes in the length of the transmission season for dengue over the period 2070–2099 for the RCP2.6-SSP1 scenario stratified by climate model.

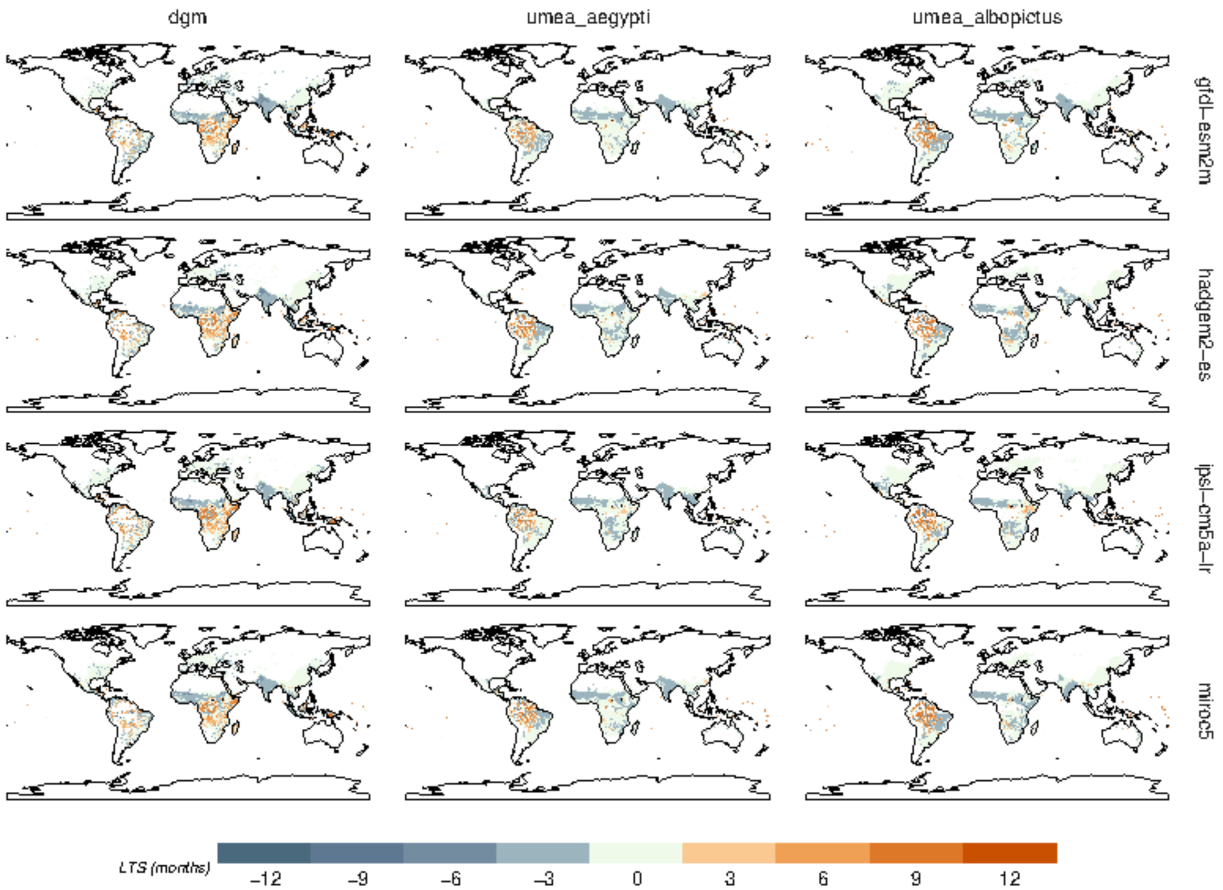

Fig A25: Simulated changes in the length of the transmission season for dengue over the period 2070–2099 for the RCP2.6-SSP2 scenario stratified by climate model.

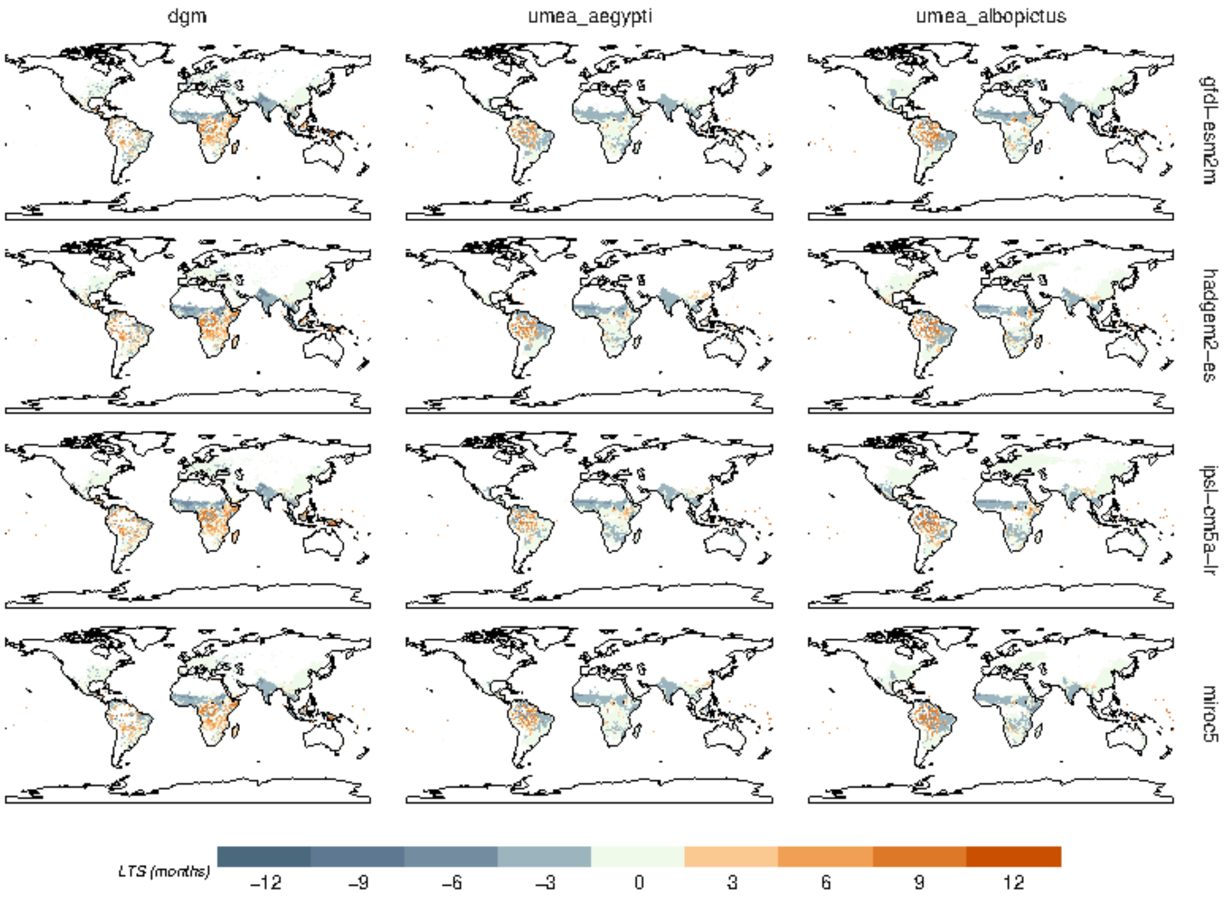

Fig A26: Simulated changes in the length of the transmission season for dengue over the period 2070–2099 for the RCP4.5-SSP2 scenario stratified by climate model.

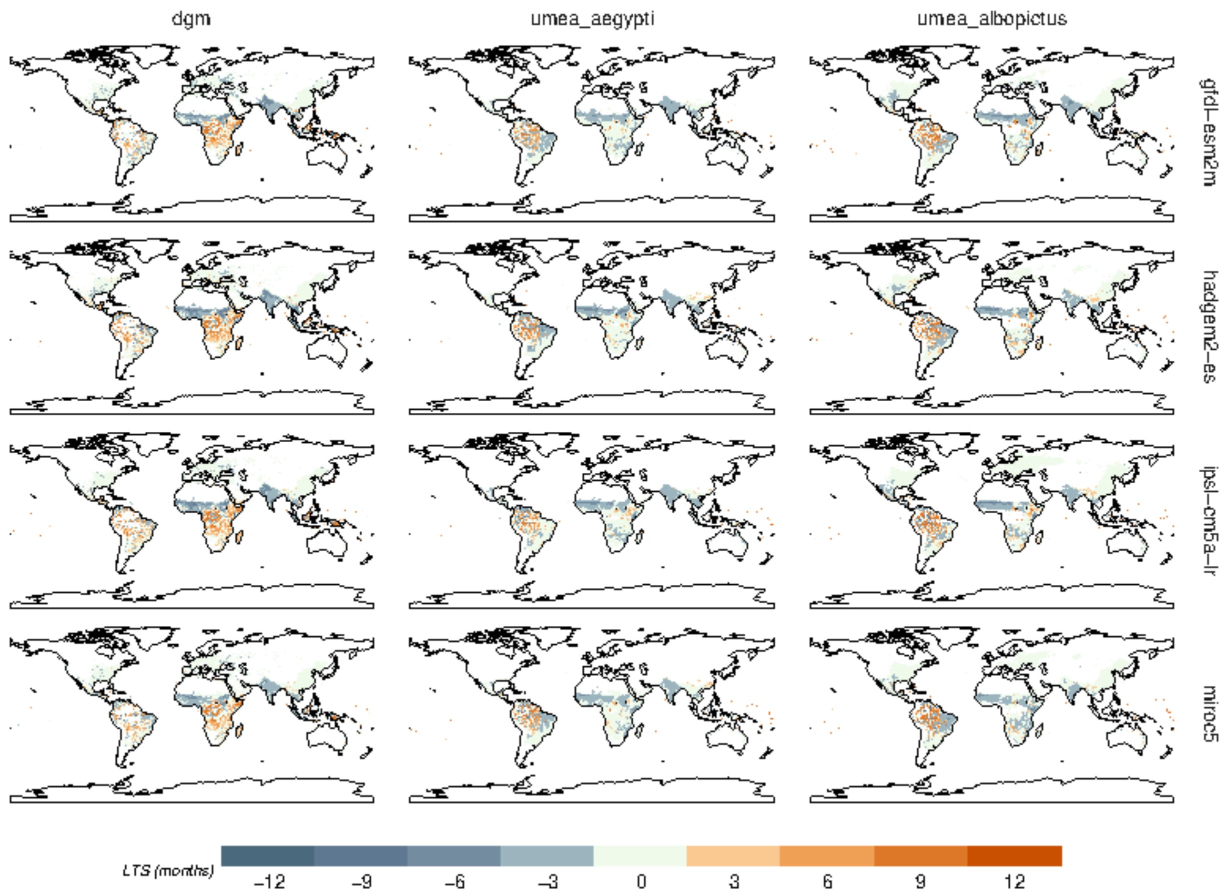

Fig A27: Simulated changes in the length of the transmission season for dengue over the period 2070–2099 for the RCP6.0-SSP2 scenario stratified by climate model.

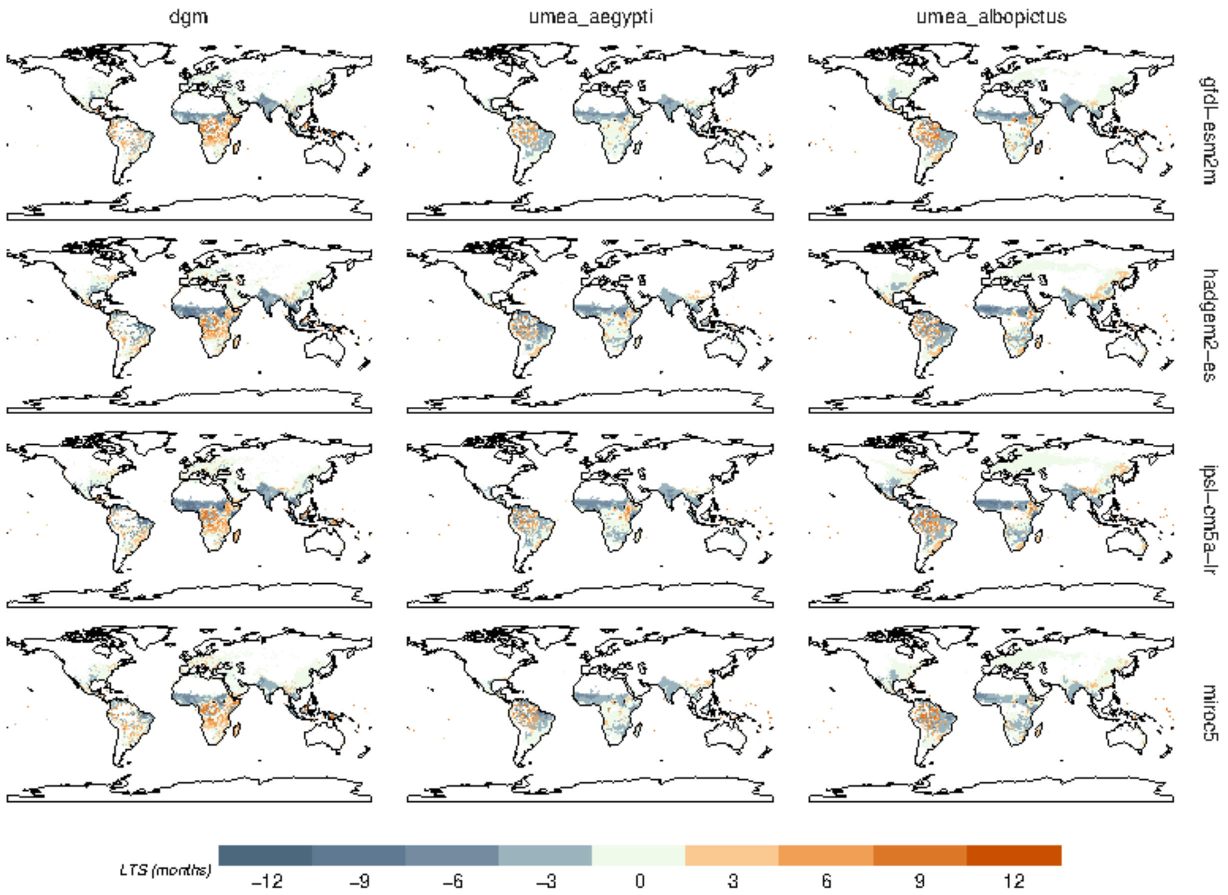

Fig A28: Simulated changes in the length of the transmission season for dengue over the period 2070–2099 for the RCP8.5-SSP2 scenario stratified by climate model.

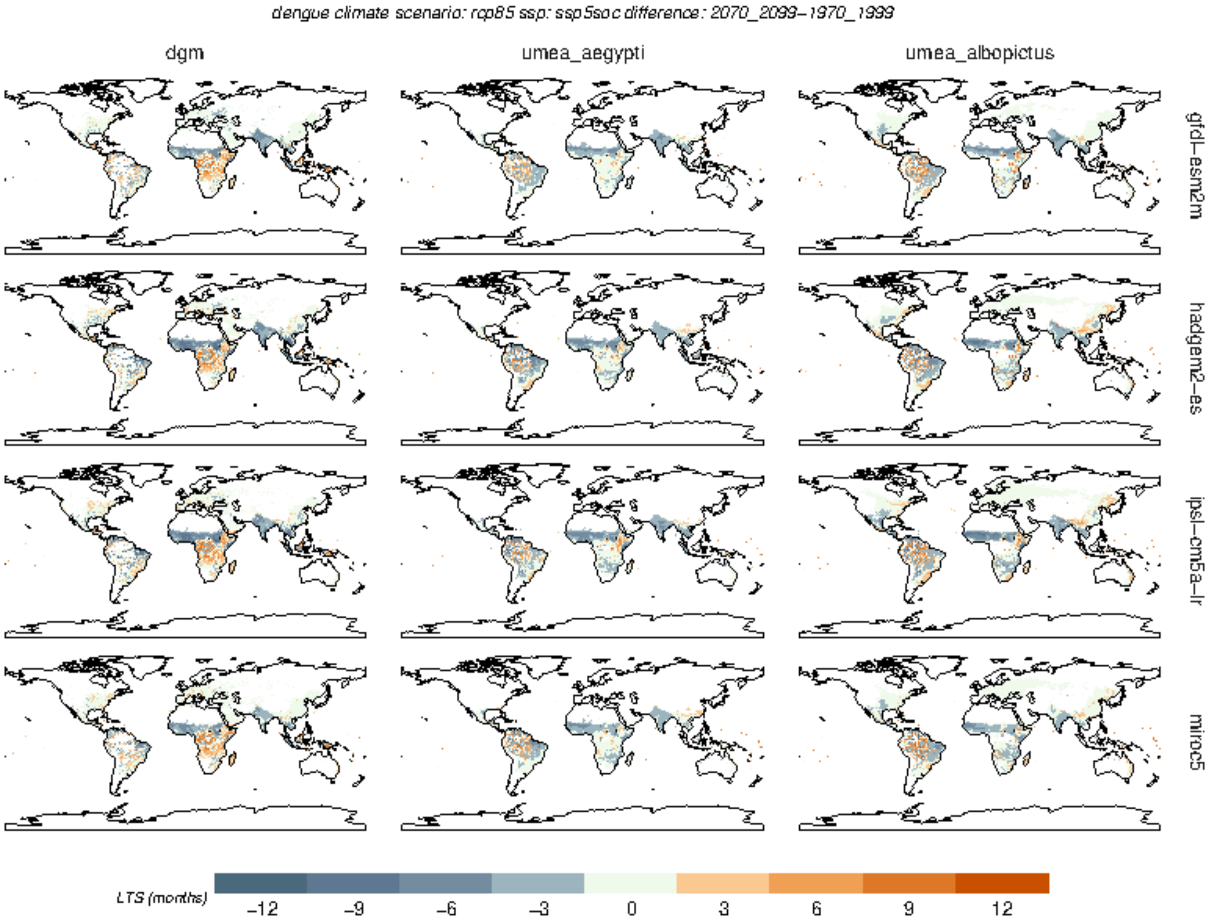

Fig A29: Simulated changes in the length of the transmission season for dengue over the period 2070–2099 for the RCP8.5-SSP5 scenario stratified by climate model.

References

1. Schulzweida U. CDO User Guide; 2019. Available from: https://doi.org/10.5281/zenodo.3539275.
2. Hijmans RJ. raster: Geographic Data Analysis and Modeling; 2020. R package version 3.3-13. Available from: https://CRAN.R-project.org/package=raster.
3. O'Neill BC, Tebaldi C, van Vuuren D, Eyring V, Friedlingstein P, Hurtt G, et al. The Scenario Model Intercomparison Project (ScenarioMIP) for CMIP6. 2016 Apr. Available from: https://doi.org/10. 5194/gmd-2016-84.
4. Tompkins AM, Thomson MC. Uncertainty in malaria simulations in the highlands of Kenya: Relative contributions of model parameter setting, driving climate and initial condition errors. PLoS One. 2018;13(9):e0200638.
5. Hay SI, Snow RW. The Malaria Atlas Project: developing global maps of malaria risk. PLoS Med. 2006;3(12):e473.
6. Gaffin SR, Rosenzweig C, Xing X, Yetman G. Downscaling and geo-spatial gridding of socio-economic projections from the IPCC Special Report on Emissions Scenarios (SRES). Global Environmental Change. 2004;14(2):105–123. Available from: https://doi.org/10.1016/j.gloenvcha.2004.02.004.
7. Rocklöv J, Tozan Y. Climate change and the rising infectiousness of dengue. Emerging Topics in Life Sciences. 2019;3(2):133–142. Available from: https://doi.org/10.1042/etls20180123.
8. Liu-Helmersson J, Brännström A, Sewe MO, Semenza JC, Rocklöv J. Estimating Past, Present, and Future Trends in the Global Distribution and Abundance of the Arbovirus Vector *Aedes aegypti* Under Climate Change Scenarios. Frontiers in Public Health. 2019;7. Available from: https://doi.org/ 10.3389/fpubh.2019.00148.
9. DiSera L, Sjödin H, Rocklöv J, Tozan Y, Súdre B, Zeller H, et al. The Mosquito, the Virus, the Climate: An Unforeseen Reunion in 2018. GeoHealth. 2020;4(8). Available from: https://doi.org/10. 1029/2020gh000253.
10. Metelmann S, Caminade C, Jones A, JM JM, Baylis M, AP Morse Journal of The Royal Society Interface. The UK's suitability for *Aedes albopictus* in current and future climates. 2019;16(152):20180761.
11. Soetaert K, Petzoldt T, Setzer RW. Solving Differential Equations in R: Package deSolve. Journal of Statistical Software. 2010;33(9):1–25. Available from: http://www.jstatsoft.org/v33/i09.
12. Liu Y, Lillepold K, Semenza JC, Tozan Y, Quam MBM, Rocklöv J. Reviewing estimates of the basic reproduction number for dengue, Zika and chikungunya across global climate zones. Environmental Research. 2020 Mar;182:109114. Available from: https://doi.org/10.1016/j.envres.2020.109114.
13. Colón-González FJ, Harris I, Osborn T, Steiner Sao Bernardo C, Peres C, Hunter P, et al. Limiting global-mean temperature increase to 1.5–2°C could reduce the incidence and spatial spread of dengue fever in Latin America. Proc Natl Acad Sci U S A. 2018;115:6243–6248.
14. Wood SN. Generalized Additive Models: An Introduction with R. 2nd ed. Chapman and Hall/CRC; 2017.
15. Tompkins AM, Ermert V. A regional-scale, high resolution dynamical malaria model that accounts for population density, climate and surface hydrology. Malaria Journal. 2013;12(1):65.
16. Asare EO, Tompkins AM, Bomblies A. A regional model for malaria vector developmental habitats evaluated using explicit, pond-resolving surface hydrology simulations. PLoS One. 2016;11(3):e0150626.
17. Laneri K, Bhadra A, Ionides EL, Bouma M, Dhiman RC, Yadav RS, et al. Forcing versus feedback: epidemic malaria and monsoon rains in northwest India. PLoS Comput Biol. 2010;6(9):e1000898.

18. Lunde TM, Korecha D, Loha E, Sorteberg A, Lindtjørn B. A dynamic model of some malaria transmitting anopheline mosquitoes of the Afrotropical region. I. Model description and sensitivity analysis. Malaria Journal. 2013;12(1):28.

19. Lutambi AM, Penny MA, Smith T, Chitnis N. Mathematical modelling of mosquito dispersal in a heterogeneous environment. Mathematical biosciences. 2013;241(2):198–216.
